# D-Peptide-Based Probe for CXCR4-Targeted Molecular Imaging and Radionuclide Therapy

**DOI:** 10.3390/pharmaceutics13101619

**Published:** 2021-10-05

**Authors:** Kaat Luyten, Tom Van Loy, Christopher Cawthorne, Christophe M. Deroose, Dominique Schols, Guy Bormans, Frederik Cleeren

**Affiliations:** 1 Laboratory for Radiopharmaceutical Research, Department of Pharmaceutical and Pharmacological Sciences, KU Leuven, 3000 Leuven, Belgium; kaat.luyten@kuleuven.be (K.L.); guy.bormans@kuleuven.be (G.B.); 2 Laboratory of Virology and Chemotherapy, Department of Microbiology, Immunology and Transplantation, KU Leuven, 3000 Leuven, Belgium; tom.vanloy@kuleuven.be (T.V.L.); dominique.schols@kuleuven.be (D.S.); 3 Nuclear Medicine and Molecular Imaging, Department of Imaging and Pathology, KU Leuven, 3000 Leuven, Belgium; christopher.cawthorne@kuleuven.be (C.C.); christophe.deroose@uzleuven.be (C.M.D.)

**Keywords:** CXCR4, PET imaging, D-peptide-based probe, cancer

## Abstract

Positron emission tomography (PET) imaging of the C-X-C chemokine receptor 4 (CXCR4) with [^68^Ga]PentixaFor has intrinsic diagnostic value and is used to select patients for personalized CXCR4-targeted radionuclide therapy with its therapeutic radiopharmaceutical companion [^177^Lu]PentixaTher. However, a CXCR4-targeting radiopharmaceutical labeled with fluorine-18 is still of high value due to its favorable characteristics over gallium-68. Furthermore, clinical results with [^177^Lu]PentixaTher are promising, but there is still room for improvement regarding pharmacokinetics and dosimetry profile. Therefore, this study aimed to develop innovative CXCR4-targeting radiopharmaceuticals, both for diagnostic and therapeutic purposes, starting from a D-amino acid-based peptide probe (DV1-k-(DV3)) that conserves high CXCR4 binding affinity after radiolabeling. AlF-NOTA-DV1-k-(DV3) showed similar in vitro binding affinity to human CXCR4 (hCXCR4) compared to [^nat^Ga]PentixaFor (half-maximal inhibitory concentration (IC_50_): 5.3 ± 0.9 nM and 8.6 ± 1.1 nM, respectively) and also binds to murine CXCR4 (mCXCR4) (IC_50_: 33.4 ± 13.5 nM) while [^nat^Ga]PentixaFor is selective for hCXCR4 (IC_50_ > 1000 nM for mCXCR4). Both the diagnostic radiotracers based on the DV1-k-(DV3) vector platform, [^18^F]AlF-NOTA-DV1-k-(DV3) and [^68^Ga]Ga-DOTA-DV1-k-(DV3), and their therapeutic companion [^177^Lu]Lu-DOTA-DV1-k-(DV3) were successfully produced in high yield, demonstrated high in vitro and in vivo stability, and have the same favorable pharmacokinetic profile. Furthermore, in wild-type mice and a hCXCR4-expressing tumor model, [^18^F]AlF-NOTA-DV1-k-(DV3) shows CXCR4-specific targeting in mCXCR4-expressing organs such as liver (mean standardized uptake value (SUV_mean_) 8.2 ± 1.0 at 75 min post-injection (p.i.)), spleen (SUV_mean_ 2.5 ± 1.0 at 75 min p.i.), and bone (SUV_mean_ 0.4 ± 0.1 at 75 min p.i., femur harboring bone marrow) that can be blocked with the CXCR4 antagonist AMD3100. However, in a hCXCR4-expressing tumor model, tumor uptake of [^18^F]AlF-NOTA-DV1-k-(DV3) was significantly lower (SUV_mean_ 0.6 ± 0.2) compared to [^68^Ga]PentixaFor (SUV_mean_ 2.9). This might be explained by the high affinity of [^18^F]AlF-NOTA-DV1-k-(DV3) toward both mCXCR4 and hCXCR4. High mCXCR4 expression in mouse liver results in a large fraction of [^18^F]AlF-NOTA-DV1-k-(DV3) that is sequestered to the liver, resulting despite its similar in vitro affinity for hCXCR4, in lower tumor accumulation compared to [^68^Ga]PentixaFor. As CXCR4 is not expressed in healthy human liver, the findings in mice are not predictive for the potential clinical performance of this novel class of CXCR4-targeting radiotracers. In conclusion, the DV1-k-(DV3) scaffold is a promising vector platform for translational CXCR4-directed research.

## 1. Introduction

The C-X-C chemokine receptor 4 (CXCR4) is a seven transmembrane G protein-coupled receptor and is involved in various inflammatory and autoimmune diseases, as well as in cancer metastasis and progression [1]. Binding of the endogenous ligand CXCL12 (also known as stromal cell-derived factor-1α (SDF-1α)) to CXCR4 leads to activation of downstream signaling pathways that exert critical functions in development (organogenesis), normal physiology as well as in disease processes [2]. The central element in these processes is the migration and retention of CXCR4-expressing cells to tissues with high SDF-1α expression. Physiologically, this includes the recruitment of stem and progenitor cells during embryogenesis, hematopoiesis and neoangiogenesis as well as leukocyte trafficking during immune response [3]. In cancer, the CXCR4-SDF-1α axis fulfills several tumor-growth supporting functions, including stimulation of tumor proliferation and angiogenesis, promotion of tumor invasiveness. Moreover, it facilitates metastasis of CXCR4-expressing tumor cells to mesenchymal stromal tissues with high SDF-1α expression such as liver, bone marrow, lungs and lymph nodes [1]. CXCR4 overexpression has been reported for multiple human tumor types, ranging from hematologic malignancies (e.g., multiple myeloma) to solid tumors, including breast, prostate, lung and colorectal cancer [4,5]. Furthermore, high CXCR4 expression is correlated with distant metastasis and poor overall and disease-free survival in patients [4].

In recent years, several CXCR4 imaging agents were developed to meet the clinical need for pre-therapeutic quantification of CXCR4 expression and to allow patient selection for CXCR4-targeted therapies. [^68^Ga]PentixaFor was translated to the clinic thanks to its outstanding properties, such as high affinity and selectivity for human CXCR4 (hCXCR4) in combination with rapid renal plasma clearance and urinary excretion, resulting in high-contrast positron emission tomography (PET) images of CXCR4-expressing tissues [6]. Based on recent clinical research, mostly performed with [^68^Ga]PentixaFor, CXCR4 is now an established target for PET imaging of hematologic tumors including non-Hodgkin lymphoma, multiple myeloma, chronic lymphocytic leukemia, acute myeloid leukemia and Waldenström macroglobulinemia [6]. CXCR4 PET imaging has intrinsic diagnostic value but can also be used as a biomarker to predict outcome and to monitor the efficacy of e.g., radiotherapy and immunotherapy [7].

Despite the good imaging properties of [^68^Ga]PentixaFor, widespread implementation in clinical practice is hampered by the limited batch production yield of current ^68^Ge/^68^Ga generators and the relatively short half-life (67.7 min) of galium-68 that challenges centralized production and distribution. However, it must be stated that larger amounts of gallium-68 can also be obtained by proton irradiation of zinc-68 targets in a cyclotron [8], but this production method is not routinely used in the clinics yet. In contrast, fluorine-18 combines several advantages such as a longer half-life of 109.8 min and the large quantities in which it can be produced with a cyclotron [9]. Attempts to make a suitable fluorine-18 derivative of [^68^Ga]PentixaFor were unsuccessful due to the pronounced sensitivity of the PentixaFor scaffold toward even minor structural modifications, leading to strongly decreased CXCR4 binding affinity [10]. Furthermore, [^177^Lu]PentixaTher, the 3-iodo-D-Tyr [1] derivative of PentixaFor, was developed as a therapeutic companion [11]. However, the 3-iodo-D-Tyr increases the lipophilicity of PentixaTher by more than one order of magnitude compared to [^68^Ga]PentixaFor. As a result, hepatic activity levels remain persistently high, up to seven days post-injection (p.i.) in humans [7]. Although [^177^Lu]PentixaTher already has been successfully used in various hematologic malignancies [7], there is still room for improvement regarding biodistribution pharmacokinetics and dosimetry profile.

In this study, we evaluated a unique CXCR4-targeting scaffold that possesses high CXCR4 binding affinity, high in vitro and in vivo stability, and good pharmacokinetic properties. We labeled the scaffold with both the diagnostic PET radionuclide fluorine-18 and therapeutic radionuclides to evaluate its use in a translational theranostic setting. We selected DV1-k-(DV3) as a potential vector molecule for the development of CXCR4-targeting radioprobes owing its unique characteristics [12]. Viral macrophage inflammatory protein-II (vMIP-II) is a viral chemokine encoded by the human herpesvirus-8 that binds CXCR4 with a high affinity (half-maximal inhibitory concentration (IC_50_) vMIP-II: 3.0 nM) to inhibit the endogenous ligand-induced calcium responses [13]. Zhou et al. designed a series of vMIP-II-derived CXCR4 antagonists composed entirely of D-amino acids, demonstrating remarkable stereochemical flexibility [14]. The bivalent peptide DV1-k-(DV3) showed high affinity for CXCR4 (IC_50_ DV1-k-(DV3): 4 nM) [12], and due to its D-amino acids composition, a high in vivo stability of the vector molecule and resulting radioprobes is expected.

**Figure 1:**
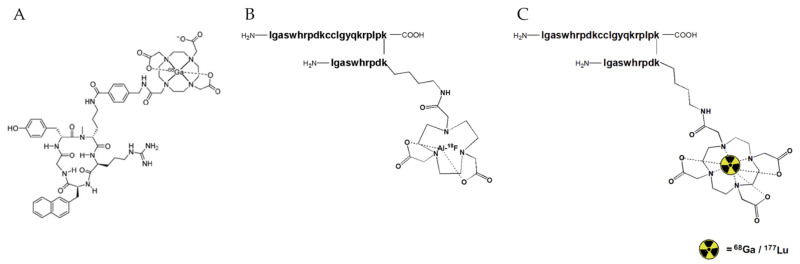
Chemical structures of radioprobes targeting the C-X-C chemokine receptor 4 (CXCR4). (**A**) [^68^Ga]PentixaFor (**B**) [^18^F]AlF-NOTA-DV1-k-(DV3) (**C**) [^68^Ga]Ga-DOTA-DV1-k-(DV3) and [^177^Lu]Lu-DOTA-DV1-k-(DV3).

DV1-k-(DV3) was site-specifically derivatized with 1,4,7-triazacyclononane-1,4,7-triacetic acid (NOTA) or 1,4,7,10-tetraraazacyclododecane-1,4,7,10-tetraacetic acid (DOTA) via the lysine residue of DV3. Next, CXCR4-binding affinity of the DV1-k-(DV3) derivatives was determined in a competitive binding assay with fluorescently labeled CXCL12. The DV1-k-(DV3) scaffold was successfully labeled using the Al^18^F-labeling method, which combines the advantages of chelator-based radiochemistry in aqueous medium with the unique properties of fluorine-18 [15]. To explore the potential of DV1-k-(DV3) as an innovative vector platform for both diagnostic and therapeutic purposes, the construct DOTA-DV1-k-(DV3) was radiolabeled with gallium-68 and the therapeutic radionuclide lutetium-177. The novel CXCR4-targeting radioprobes (Figure 1) were further evaluated in wild-type mice and a hCXCR4-expressing tumor mouse model. Results were compared with the clinically established radiopharmaceutical [^68^Ga]PentixaFor.

## 2. Materials and Methods

### 2.1. Synthesis and Radiolabeling

Resin-bound DV1-k-(DV3) [12] (Pepmic Co., Ltd. (Suzhou, China)) was site-specifically derivatized with 2-(4,7-bis(2-(tert-butoxy)-2-oxoethyl)-1,4,7-triazonan-1-yl)acetic acid (NOTA(tBu)_2_, CheMatech, Dijon, France) or 2-(4,7,10-tris(2-(tert-butoxy)-2-oxoethyl)-1,4,7,10-tetraazacyclododecan-1-yl)acetic acid (DOTA(tBu)_3_, CheMatech, Dijon, France) onto the ε-amino moiety of the *C*-terminal DV3 lysine under standard peptide coupling conditions using (2-(7-Aza-1H-benzotriazole-1-yl)-1,1,3,3-tetramethyluronium hexafluorophosphate (HATU) and *N*-ethyldiisopropylamine (DIPEA) to obtain NOTA-DV1-k-(DV3) and DOTA-DV1-k-(DV3), after deprotection and HPLC purification, respectively. For further upscaling, all DV1-k-(DV3) derivatives were purchased from Pepmic Co., Ltd. AlF-, ^nat^Ga-, ^nat^Lu-, ^nat^Bi- and La-complexes were synthesized in analogy to previously published protocols [16]. DV1-k-(DV3) was oxidized in metal-free water (20% DMSO) and reacted for 36 h at room temperature. Complete oxidation of both cysteines in the DV1 sequence was confirmed by LC-HRMS analysis (analytical data in the Supplementary Materials). NOTA-DV1-k-(DV3) was labeled using the Al^18^F-method in an AllInOne^®^ synthesis module (Trasis, Ans, Belgium) [17]. DOTA-DV1-k-(DV3) was labeled with galium-68 in an automated SCINTOMICS GRP module (Scintomics, Fuerstenfeldbruck, Germany) and via a manual procedure with lutetium-177. Quality control of all radioprobes was performed using radioHPLC. A detailed synthesis protocol, analytical data for the peptides and a detailed description of the Al^18^F-, ^68^Ga- and ^177^Lu-labeling conditions and radioHPLC conditions, including recovery, are provided in the Supplementary Materials.

### 2.2. Analysis

#### 2.2.1. LC-HRMS

A Dionex Ultimate 3000 UPLC System (Thermo Fisher Scientific, Waltham, MA, USA) coupled in series to a UV detector, a 3-inch NaI(Tl) radioactivity detector, and an ultra-high resolution time-of-flight mass spectrometer with electrospray ionization (ESI) (MaXis Impact, Bruker, Bremen, Germany) was used for analysis of all non-radioactive DV1-k-(DV3) constructs. Solvent A (water, 0.1% HCOOH) and solvent B (acetonitrile, 0.1% HCOOH), flow rate 0.6 mL/min, Acquity UPLC BEH C_18_ 1.7 µm 2.1 × 50 mm column (Waters Corporation, Milford, MA, USA). The elution gradient was: 0–2 min: 95% A; 2–10 min: from 95% A to 5% A; 10–12 min: 95% A. UV monitoring of the eluate was performed at 220 nm. For deconvolution analysis of the raw mass spectral data, the software program DataAnalysis (Bruker Daltonik, Bremen, Germany) was used. Calculated average neutral molecular ion mass values were obtained using Compass IsotopePattern (version 3.2, Bruker Daltonik, Bremen, Germany) software.

#### 2.2.2. iTLC

Instant thin-layer liquid chromatography papers (iTLC-SG, Varian, Diegem, Belgium) were developed in an elution chamber using 0.5 M sodium citrate pH 5.5. The strips were analyzed using phosphor storage screens (super-resolution screen, Perkin Elmer, Waltham, MA, USA). Screens were read in a Cyclone Plus system (Perkin Elmer, Waltham, MA, USA), and images were analyzed using Optiquant software (Perkin Elmer, Waltham, MA, USA) or by an automated gamma counter which contained a 3-inch NaI(Tl) well crystal linked to a multichannel analyzer (2480 Wizard^2^, Perkin Elmer, Waltham, MA, USA). For quantification, counts were corrected for background, counter dead time and physical decay during counting.

#### 2.2.3. RadioHPLC

RadioHPLC was performed using an Elite LaChrom VWR Hitachi pump L-2130 connected to an Elite LaChrom VWR Hitachi UV detector L-2400, an Alltech Elite On-line Degassing System from Grace Davison Discovery Sciences (Lokeren, Belgium) and a GABI Star from Elysia Raytest (Angleur, Belgium) as a radioactivity-HPLC-flow monitor. An Xbridge (3.5 µm, 3 × 100 mm) column (Waters Corporation, Milford, MA, USA) (used for analysis of [^68^Ga]Ga-DOTA-DV1-k-(DV3), [^68^Ga]PentixaFor and [^177^Lu]Lu-DOTA-DV1-k-(DV3) or PRP-1 (1.5 µm, 4.1 × 150 mm) column (Hamilton, Bonaduz, Graubünden, Switzerland) (used for analysis of [^18^F]AlF-NOTA-DV1-k-(DV3)) with a flow rate of 0.8 mL/min was used. Acetonitrile (0.1% trifluoroacetic acid (TFA)) in water (0.1% TFA) was used as the mobile phase with the following multistep gradient: 0–20 min, 5–95% acetonitrile (0.1% TFA); 20–25 min, 95–5% acetonitrile (0.1% TFA); 25–30 min 5% acetonitrile (0.1% TFA).

#### 2.2.4. Animals and Cell Lines

For in vitro experiments, the following cell lines were used: hCXCR4-expressing Jurkat human T-cell leukemia cells, U87 human glioblastoma cells stably transfected with hCXCR4 or mCXCR4 (U87.hCXCR4 and U87.mCXCR4, respectively). Jurkat cells were cultured in RPMI-1640 medium containing 10% fetal bovine serum (FBS) and 2 mM glutamine. U87 cells were maintained in Dulbecco modified Eagle medium, with L-glutamine and 10% FBS supplemented by a 2 µg/mL concentration of puromycin. All cell lines were maintained at 37 °C in a humidified atmosphere with 5% CO_2_. Female NMRI mice (wild-type, 5 weeks-old, Envigo RMS BV, Venray, The Netherlands) and female CB17-SCID mice (6–8 weeks-old, Charles River Laboratories, Sulzfeld, Germany) were used for in vivo evaluation. Animals were housed in individually ventilated cages in a thermo-regulated (~22 °C), humidity-controlled facility under a 12 h-12 h light-dark cycle, with access to food and water ad libitum. All animal experiments were conducted according to the Belgian code of practice for the care and the use of animals, after approval from the university animal ethics committee.

### 2.3. In Vitro Evaluation

#### 2.3.1. Determination of hCXCR4/mCXCR4 Affinity

Competition binding studies (IC_50_) were performed as described [18] using either Jurkat cells and human CXCL12^AF647^ (Almac, UK) as fluorescent ligand or U87.mCXCR4 cells and murine CXCL12^AF647^ (Almac, UK) as fluorescent ligand. For each DV1-k-(DV3)-based probe, [^nat^Ga]PentixaFor and AMD3100, the dose-dependent ability to inhibit binding of fluorescently labeled CXCL12 to CXCR4 was determined by flow cytometry. IC_50_-values were calculated using nonlinear regression (four parameters) in GraphPad Prism 9 (Graph Pad Software, San Diego, CA, USA) based on three biological replicates for each concentration tested.

#### 2.3.2. Stability Assessment (Supplementary Materials)

Formulated solutions of [^18^F]AlF-NOTA-DV1-k-(DV3) and [^68^Ga]Ga-DOTA-DV1-k-(DV3) were evaluated by radioHPLC 5 h and 2 h after radiosynthesis, respectively. In addition, 5-fold dilutions in human serum (H4522 human male AB plasma, USA origin, sterile-filtered, Sigma Aldrich, Saint Louis, MO, USA) were incubated at 37 °C for 2 h and analyzed using radioHPLC.

A formulated solution of [^177^Lu]Lu-DOTA-DV1-k-(DV3) was evaluated by radioHPLC after 9 days of storage at 4 °C. A 5-fold dilution of [^177^Lu]Lu-DOTA-DV1-k-(DV3) in human serum (H4522 human male AB plasma, USA origin, sterile-filtered, Sigma Aldrich, Saint Louis, MO, USA) was incubated at 37 °C for 9 days and analyzed by radioHPLC.

#### 2.3.3. Binding and Internalization Assay

Cellular uptake and internalization of [^18^F]AlF-NOTA-DV1-k-(DV3), [^68^Ga]Ga-DOTA-DV1-k-(DV3) and [^177^Lu]Lu-DOTA-DV1-k-(DV3) (11, 17 and 7 nM respectively) into U87.hCXCR4 cells (2.5 × 10^5^ cells) were investigated and compared with [^68^Ga]PentixaFor (6 nM) as control. Non-specific uptake was determined in the presence of 100 μM AMD3100. Briefly, U87.hCXCR4 (2.5 × 10^6^ cells) were incubated with the radioligand of interest (*n* = 3, 185 kBq) for 1 h at 37 °C in Hank’s Balanced Salt Solution (HBSS (1×), 20 mM HEPES, 0.2% BSA) in the presence or absence of AMD3100 (100 µM, *n* = 3). After incubation, the supernatant was removed and combined with 3 mL of ice-cold PBS used for rinsing the cells (unbound fraction). Subsequently, cells were incubated twice with 50 mM Glycine-HCl pH 2.8 for 5 min at room temperature, followed by one washing step with ice-cold PBS (membrane-bound fraction). Then, cells were lysed using 350 µL of reagent A100 (Chemometec, Allerod, Denmark). Reagent B (Chemometec, Allerod, Denmark) was used for rinsing the wells and to quench the lysing of the cells. Quantification of the amount of bound and internalized activity was performed using an automated gamma counter. The number of cells per well was counted using an automated counting device (NucleoCounter^®^ NC-100™, Chemometec, Allerod, Denmark). Results were expressed as percentage of applied radioactivity bound to 2.5 × 10^5^ cells.

### 2.4. In Vivo Evaluation

#### Stability Assessment

Wild-type mice were anesthetized (2.5% isoflurane in O_2_ at 1 L/min flow rate) and intravenously injected with [^18^F]AlF-NOTA-DV1-k-(DV3) (4.5–10 MBq). Plasma samples were obtained by decapitation 15 min p.i. (*n* = 2). Blood was collected in EDTA tubes and centrifuged for 5 min at 3000× *g*. Subsequently, plasma was removed and diluted (1:2) with acetonitrile to precipitate all plasma proteins. After centrifugation (5 min at 3000× *g*), the supernatant was diluted (1:10) with water and filtered (0.22 µm) before injection on radioHPLC. Urine samples were collected 75 min p.i. (*n* = 3) by gently pressing on the bladder. The collected urine was filtered (0.22 µm) before injection on radioHPLC. Due to low radioactivity concentration in the plasma samples, 30 fractions of 30 s per plasma sample were collected. Radioactivity in the fractions was determined in an automated gamma counter.

### 2.5. Ex Vivo Autoradiography

Wild-type mice were anesthetized (2.5% isoflurane in O_2_ at 1 L/min flow rate) and intravenously injected with [^18^F]AlF-NOTA-DV1-k-(DV3) and [^68^Ga]Ga-DOTA-DV1-k-(DV3) (3.5–4 MBq). After 75 min, mice were sacrificed by decapitation. Organs of interest were quickly excised and snap frozen in 2-methylbutane (−40 °C). Next, 20 μm sections of liver, kidney and spleen were obtained using a cryotome (Shandon cryotome FSE; Thermo Fisher Scientific, Waltham, MA, USA) and these were mounted on adhesive microscope slides (Superfrost Plus; Thermo Fisher Scientific, Thermo Fisher Scientific, Waltham, MA, USA) after which they were exposed to a phosphor storage screen (super-resolution screen; Perkin Elmer, Waltham, MA, USA) overnight. Screens were read in a Cyclone Plus system (Perkin Elmer, Waltham, MA, USA), and images were analyzed using OptiQuant software (Perkin Elmer, Waltham, MA, USA). The remaining excised tissue was further sectioned in 20 μm slices and stored at −20 °C.

### 2.6. Preparation of Tumor Xenograft Model

All animal procedures were approved by the KU Leuven ethical review board (ethical approval reference P012-2020) and were carried out in accordance with Directive 2010/63/EU and reported according to the ARRIVE guidelines [19]. The number of animals included in the study was based on previous reports where an approximately 70% difference was seen between naïve and blocked animals in CXCR4 expressing organs [20]. Detection of such difference therefore required 3 animals per group (power = 0.95 and *α* = 0.05) assuming a 20% standard deviation within the groups.

The subcutaneous tumor xenograft model was prepared using a published procedure [21]. Briefly, 1 × 10^6^ U87.hCXCR4 cells mixed with Cultrex (1:1; Cultrex Basement Membrane Extract, R&D systems, Minneapolis, MN, USA) were implanted subcutaneously into the right shoulder of female 10-week-old SCID mice (CB17.Cg-Prkdc<scid>Lyst<bg-J>/Crl; Charles River Laboratories, Sulzfeld, Germany). After an average of four weeks, the animals were used in biodistribution and PET/computed tomography (CT) imaging studies. Tumor volumes ranged from 150–574 mm^3^ (measured by caliper, h × l × w), while the tumor mass-to-body weight ratio was 0.14–2.34%.

All animals included in the study were randomly selected among the in-house bred mice of the correct age. There were no exclusion criteria, and all subsequent studies and analyses were conducted unblinded.

### 2.7. PET/CT Imaging and Biodistribution Study

The in vivo pharmacokinetics of the selected radioligands were first evaluated in wild-type mice by performing a whole-body biodistribution study. 75 min p.i. of [^68^Ga]PentixaFor (2 MBq/0.1 nmol PentixaFor/mouse), [^18^F]AlF-NOTA-DV1-k-(DV3) (1.5–3.0 MBq/0.2–0.4 nmol DV1-k-(DV3)/mouse), [^68^Ga]Ga-DOTA-DV1-k-(DV3) (1.5–3.0 MBq/0.2–0.4 nmol DV1-k-(DV3)/mouse) or [^177^Lu]Lu-DOTA-DV1-k-(DV3) (0.07 MBq/1 nmol DV1-k-(DV3)/mouse), in the presence or absence of AMD3100 (5 mg/kg/mouse), mice were sacrificed. Blood and major organs were collected in tared tubes and weighed. Quantification of radioactivity in blood, organs, and other body parts was performed using an automated gamma counter equipped with a 3-inch NaI(Tl) well crystal coupled to a multichannel analyzer, mounted in a sample changer (2480 Wizard^2^, Perkin Elmer, Waltham, MA, USA). Counts were corrected for background radiation, physical decay and counter dead time. Results are presented as standardized uptake values (SUV; tissue activity concentration (MBq/g)/[injected dose (MBq)/body weight (g)]).

Small animal whole-body PET imaging was performed by intravenously administering [^18^F]AlF-NOTA-DV1-k-(DV3) (1.5–3.0 MBq/0.2–0.4 nmol DV1-k-(DV3)/mouse) or [^68^Ga]PentixaFor (2 MBq/0.1 nmol PentixaFor/mouse) in the presence or absence of AMD3100 (5 mg/kg/mouse) or DV1-k-(DV3) (7, 14, 23 or 57 nmol/mouse) into wild-type and U87.hCXCR4 tumor-bearing mice. Dynamic PET images were acquired for 60 min immediately after intravenous injection using a beta-cube PET scanner (Molecubes, Gent, Belgium). The mice were kept under gas anesthesia during the entire procedure (2.5% isoflurane in O_2_ at 1 L/min flow rate), with temperature and respiration monitored throughout. After PET scanning, a CT image was acquired for anatomic coregistration with an X-cube CT scanner (Molecubes, Gent, Belgium), using the ‘general’ protocol with the following parameters: 50 kVp, 480 exposures, 85 ms/projection, 100 μA tube current, rotation time 60 s. After scanning, mice were sacrificed 75 min p.i. and a whole-body biodistribution study was performed.

### 2.8. Image Processing and Analysis

PET data were histogrammed into 14 frames (4 × 15 s, 4 × 1 min, 1 × 5 min, 5 × 10 min) and reconstructed into a 192 × 192 image matrix with 0.4 mm voxels using 30 iterations the native MLEM algorithm with corrections for randoms, scatter, attenuation and decay. CT Data were reconstructed using a regularized statistical (iterative) image reconstruction algorithm using non-negative least squares, using an isotropic 200 μm voxel size and scaled to Hounsfield Units (HUs) after calibration against a standard air/water phantom. Using PFUS v4.0 to display the fused PET-CT image (PMOD Technologies GmbH, Zürich, Switzerland), volumes of interest were manually drawn over the tumor while a sphere of 3 mm diameter was placed over the left lobe of the liver. Radiotracer uptake at 60 min, expressed as SUV_mean_, was the outcome measure.

### 2.9. Statistical Analysis

Quantitative data are expressed as mean ± standard deviation (SD) unless stated otherwise. Means were compared using the independent-samples *t*-test (*T*-test: two-sample assuming unequal variances in Microsoft Excel). Values were considered statistically significant for *p* < 0.05.

## 3. Results & Discussion

### 3.1. In Vitro Evaluation

**Synthesis and IC_50_ determination.** DV1 and DV3 are two synthetic peptides composed entirely of D-amino acids, derived from the *N*-terminus of vMIP-II. DV1 and DV3 consist out of 21 and 10 D-amino acid residues respectively [14]. The peptides showed moderate to high CXCR4 binding affinity (IC_50_ DV1: 32 nM, IC_50_ DV3: 439 nM) using a CXCR4-specific mAb 12G5 competing binding assay [14]. Next, the same group used a bivalent ligand approach to link DV1 and DV3, generating the dimeric peptide DV1-k-(DV3). This bivalent peptide showed very high affinity for CXCR4 (IC_50_ DV1-k-(DV3): 4 nM) and lack of binding to CXCR7 [12].

First, we successfully confirmed previously reported IC_50_-values of DV1, DV3 and DV1-k-(DV3) for hCXCR4 in an in vitro binding assay (Table 1) and we assessed the stability of the bivalent peptide DV1-k-(DV3) in standard labeling conditions (aqueous buffer pH 4, 1 h at 95 °C). No degradation products were detected by LC-HRMS analysis, showing the potential to radiolabel the peptide using these standard labeling conditions.

DV1-k-(DV3) was then site-specifically derivatized with the Al^18^F-chelator NOTA and the ^68^Ga- and ^177^Lu-chelator DOTA onto the ε-amino moiety of the *C*-terminal DV3 lysine. Constructs were purified using HPLC, lyophilized and characterized using LC-HRMS (Figures S2–S5). To explore the broad applicability of DV1-k-(DV3) as a vector molecule for CXCR4-targeted molecular imaging and radionuclide therapy, DV1-k-(DV3) derivatives were complexed with a range of natural isotopes of clinically relevant β^+^- (fluorine-18, gallium-68), β^−^- (lutetium-177) and α-emitters (actinium-225, bismuth-213). As a surrogate for actinium-225, we used lanthanum as no stable isotopes of actinium are available. Analysis of all constructs are provided in the Supplementary Materials (Figures S6–S15). Having the possibility to label the therapeutic radiopharmaceutical with an α-emitting and β^-^-emitting radionuclide allows therapy of a wide range of tumor sizes. AlF-NOTA-DV1-k-(DV3), [^nat^Ga]Ga-DOTA-DV1-k-(DV3), [^nat^Lu]Lu-DOTA-DV1-k-(DV3), [^nat^Bi]Bi-DOTA-DV1-k-(DV3) and La-DOTA-DV1-k-(DV3) were evaluated for in vitro hCXCR4-binding affinity in Jurkat cells, a human T-cell leukemia cell line expressing endogenous CXCR4. [^nat^Ga]PentixaFor and AMD3100, a known CXCR4 antagonist, were used as reference compounds (Table 1).

All DV1-k-(DV3) constructs showed similar in vitro binding affinity to hCXCR4 as [^nat^Ga]PentixaFor (nanomolar range). These findings confirm the remarkable stereochemical flexibility of the peptide core toward structural modification of the C-terminal end of the amino acid sequence. Previously, it has been shown that the *N*-terminal end of vMIP-II and its derivatives is essential for binding to CXCR4 as labeling with fluorescein at the C-terminus of DV1 did not affect affinity and ability to inhibit HIV-1 entry [22].

Another interesting finding is the slightly lower affinity of the empty chelator-constructs (NOTA-DV1-k-(DV3) and DOTA-DV1-k-(DV3)) for hCXRC4. The main binding mechanism of most CXCR4 antagonists is the interaction of positively charged moieties with the negatively charged binding pockets of CXCR4 [23]. The latter might be an explanation for the increase in IC_50_-value of the empty chelator derivatives, of which the carboxylic groups are deprotonated under physiological conditions and therefore could be repulsed by the negatively charged residues in the binding pockets of the receptor.

For the appropriate interpretation of preclinical biodistribution and imaging studies in mouse models, such as the U87.hCXCR4 tumor-bearing mice in this study, knowledge of the species dependence of target binding is of utmost importance. Consequently, the affinity of DV1-k-(DV3) and AlF-NOTA-DV1-k-(DV3) for murine CXCR4 (mCXCR4) were also determined and compared with [^nat^Ga]PentixaFor, with AMD3100 as a reference. As summarized in Table 2, DV1-k-(DV3) and its AlF-NOTA-complex have affinity for both hCXCR4 and mCXCR4, allowing translational research. In contrast, [^nat^Ga]PentixaFor only binds with high affinity to hCXCR4. This needs to be taken into account when comparing the biodistribution properties of [^18^F]AlF-NOTA-DV1-k-(DV3) and [^68^Ga]PentixaFor in a preclinical setting.

**Radiolabeling, quality control and in vitro stability.** [^18^F]AlF-NOTA-DV1-k-(DV3) was successfully produced in high yield (26 ± 6% (decay-corrected, activity in reactor/activity final batch of purified product, *n* = 6), radiochemical purity (RCP) > 98%, 11.5 ± 2.5 MBq/nmol, (*n* = 3)) via the cGMP-compatible [^18^F]AlF-NOTA labeling method previously developed for [^18^F]AlF-NOTA-octreotide [16]. The production was performed in a cassette-based automated AllinOne^®^ synthesis (Trasis) module. The high batch activity and longer half-life of fluorine-18 compared to gallium-68 allows transportation to remote hospitals without an on-site cyclotron and a larger time window between injection and scanning up to 6 h, which is limited for [^68^Ga]PentixaFor. Quality control (Figure S19) was performed with radioHPLC using a polymer-based Hamilton PRP-1 column and a buffer system of water and acetonitrile (+0.1% TFA). We opted to use a polymer-based column as in these acidic conditions, [^18^F]HF that is formed might lead to low recovery on silica-based reversed phase HPLC columns and thus overestimation of the radiochemical yield [24]. As expected, high recovery (> 95%) was observed for [^18^F]F^-^ and ([^18^F]AlF)^2+^ using the Hamilton PRP-1 column.

[^68^Ga]Ga-DOTA-DV1-k-(DV3) (radiochemical yield (RCY) 47 ± 6% (decay-corrected, activity in reactor/activity final batch of purified product, *n* = 8), RCP > 98%, 8.9 ± 1.2 MBq/nmol (*n* = 5), Figure S20) and [^68^Ga]PentixaFor (RCY 72 ± 11% (decay-corrected, activity in reactor/activity final batch of purified product, *n* = 5), RCP > 98%, 29.2 ± 3.7 MBq/nmol (*n* = 3), Figure S21) were produced using a SCINTOMICS GRP module in combination with a disposable GMP grade cassette system following a well-established standard labeling procedure. Labeling of DOTA-DV1-k-(DV3) with [^177^Lu]LuCl_3_ was carried out manually (RCY > 98% (*n* = 3), RCP > 98%, Figure S22). [^18^F]AlF-NOTA-DV1-k-(DV3) and [^68^Ga]Ga-DOTA-DV1-k-(DV3) were stable in vitro both in formulation buffer (0.9% NaCl, 0.58% Na Asc, 8% EtOH: 5 h > 98% and 2 h > 98%, respectively) and human serum (2 h at 37 °C: > 97% and > 90%, respectively) as determined with radioHPLC. (Figures S23 and S24) [^177^Lu]Lu-DOTA-DV1-k-(DV3) remained intact both in the formulation buffer (0.9% NaCl, 0.58% Na Asc: > 98%) and human serum (37 °C) (> 98%) even after 9 days of incubation. (Figure S25)

**Membrane binding and internalization.** Membrane binding and internalization of [^18^F]AlF-NOTA-DV1-k-(DV3), [^68^Ga]Ga-DOTA-DV1-k-(DV3) and [^177^Lu]Lu-DOTA-DV1-k-(DV3) on U87.hCXCR4 cells (2.5 × 10^5^ cells) were investigated in the absence or presence of AMD3100 (100 µM) and compared with [^68^Ga]PentixaFor (Table 3). The total cell-bound fraction (membrane-bound + internalized fraction) of the DV1-k-(DV3) derivatives is in the same order but slightly lower than for [^68^Ga]PentixaFor. In the presence of the structurally unrelated CXCR4 antagonist AMD3100, significant less binding was observed for all tested compounds, indicating CXCR4 specific binding. The internalized fraction of the DV1-k-(DV3) derivatives was slightly higher than for [^68^Ga]PentixaFor. Both vector molecules, PentixaFor and DV1-k-(DV3), are antagonists of hCXCR4, which are not known to be internalized by endocytosis following binding to the receptor. The higher percentage of internalization in this in vitro binding assay could be explained by uncomplete removal of the membrane-bound fraction during acidic washing steps. When using an excess of a known CXCR4 antagonist, AMD3100, cellular uptake of [^68^Ga]PentixaFor was completely blocked but only limited blocking (70–89%) was observed for our three DV1-k-(DV3) radioligands.

### 3.2. In Vivo Evaluation

**Figure 2:**
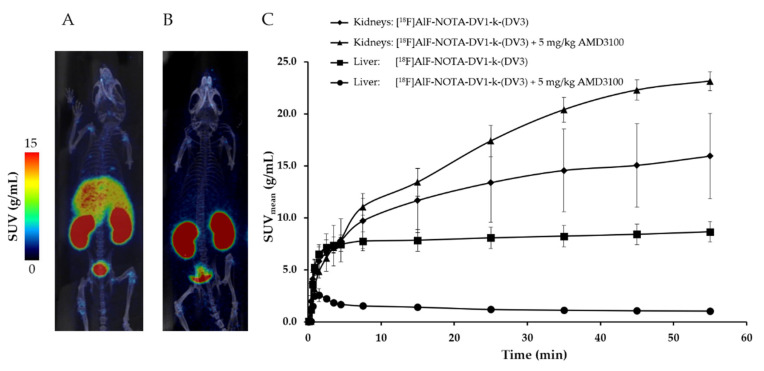
(**A**,**B**) Positron emission tomography (PET)/computed tomography (CT) imaging in wild-type mice. Averaged maximum intensity projection (MIP) image (50–60 min post-injection (p.i.)): (**A**) [^18^F]AlF-NOTA-DV1-k-(DV3) (*n* = 5), (**B**) [^18^F]AlF-NOTA-DV1-k-(DV3) + 5 mg/kg AMD3100 (*n* = 2). (**C**) Time-activity curves (TACs) expressed in mean standardized uptake value (SUV_mean_) (SUV averaged over organ volume) for kidneys and liver. All data are expressed as mean ± SD.

**Biodistribution in wild-type mice.** [^18^F]AlF-NOTA-DV1-k-(DV3) was first evaluated in vivo in wild-type mice to determine organ retention and clearance pathways of the radiotracer. During a 0–60 min p.i. dynamic PET scan, immediately after intravenously injection, fast clearance and high liver and kidney uptake was observed (Figure 2A). When co-injecting the CXCR4 antagonist AMD3100 (5 mg/kg) (Figure 2B), liver retention was completely blocked suggesting CXCR4-specific binding of [^18^F]AlF-NOTA-DV1-k-(DV3) to mCXCR4 in the liver. Furthermore, kidney uptake increased with a factor of two (Figure 2C), which can be explained by the increased radioligand blood concentration when blocking CXCR4-specific liver uptake. This in vivo biodistribution study confirmed the high in vitro binding affinity (Table 2) of AlF-NOTA-DV1-k-(DV3) for mCXCR4 and shows that [^18^F]AlF-NOTA-DV1-k-(DV3) has good pharmacokinetic properties. The fast blood clearance of [^18^F]AlF-NOTA-DV1-k-(DV3) via the renal route results in a high target-to-background signal at 50–60 min p.i. Renal clearance is the preferred clearance route, as hepatobiliary clearance of radiotracers gives high background signals in the abdomen and will decrease the signal-to-background ratio of CXCR4-expressing tumors or inflamed tissues in the abdomen.

**Figure 3:**
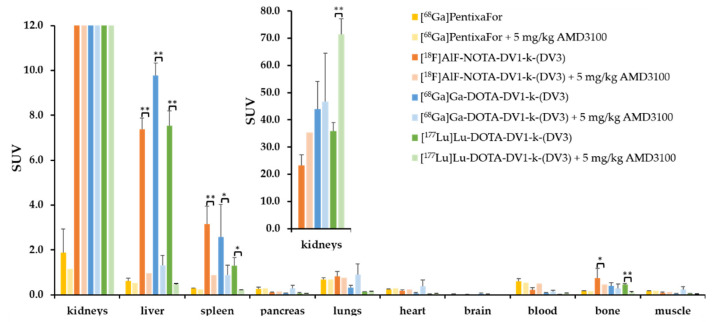
Ex vivo biodistribution in wild-type mice 75 min p.i. (expressed in SUV). [^68^Ga]PentixaFor (*n* = 3), [^68^Ga]PentixaFor + 5 mg/kg AMD3100 (*n* = 1), [^18^F]AlF-NOTA-DV1-k-(DV3) (*n* = 5), [^18^F]AlF-NOTA-DV1-k-(DV3) + 5 mg/kg AMD3100 (*n* = 2), [^68^Ga]Ga-DOTA-DV1-k-(DV3) (*n* = 4), [^68^Ga]Ga-DOTA-DV1-k-(DV3) + 5 mg/kg AMD3100 (*n* = 4), [^177^Lu]Lu-DOTA-DV1-k-(DV3) (*n* = 3) and [^177^Lu]Lu-DOTA-DV1-k-(DV3) + 5 mg/kg AMD3100 (*n* = 3). All data are expressed as mean ± SD. Statistical analysis independent-samples *t*-test. * *p* < 0.05, ** *p* ≤ 0.001.

As the goal of this study is to design a new vector platform for CXCR4-targeted molecular imaging and radionuclide therapy, in vivo pharmacokinetics of the diagnostic radiotracers, [^18^F]AlF-NOTA-DV1-k-(DV3) and [^68^Ga]Ga-DOTA-DV1-k-(DV3) were investigated and compared with those of the therapeutic radioligand, [^177^Lu]Lu-DOTA-DV1-k-(DV3) (Figure 3, Table S2). All three DV1-k-(DV3)-based radioligands have similar pharmacokinetic properties, fast blood and renal clearance and show CXCR4-specific uptake in mCXCR4-expressing organs at 75 min p.i. such as liver (SUV_mean_ 7.4 ± 0.5, 9.8 ± 0.6 and 7.5 ± 0.7 for [^18^F]AlF-NOTA-DV1-k-(DV3), [^68^Ga]Ga-DOTA-DV1-k-(DV3) and [^177^Lu]Lu-DOTA-DV1-k-(DV3) respectively), spleen (SUV_mean_ 3.2 ± 0.8, 2.6 ± 1.4 and 1.3 ± 0.4 for [^18^F]AlF-NOTA-DV1-k-(DV3), [^68^Ga]Ga-DOTA-DV1-k-(DV3) and [^177^Lu]Lu-DOTA-DV1-k-(DV3) respectively), and bone (SUV_mean_ 0.7 ± 0.4, 0.4 ± 0.1 and 0.5 ± 0.04 for [^18^F]AlF-NOTA-DV1-k-(DV3), [^68^Ga]Ga-DOTA-DV1-k-(DV3) and [^177^Lu]Lu-DOTA-DV1-k-(DV3) respectively, femur harboring bone marrow), resulting in high target-to-background ratio and good-contrast PET images. Ex vivo autoradiography of kidney, liver and spleen 75 min p.i. of [^18^F]AlF-NOTA-DV1-k-(DV3) or [^68^Ga]Ga-DOTA-DV1-k-(DV3) with or without co-injection of 5 mg/kg AMD3100 confirmed specific binding of both radiotracers to mCXCR4-expressing tissues and increased kidney accumulation in blocking conditions (Figure S26).

Intense accumulation of radioactivity in the kidneys was also observed. High kidney retention of small positively charged radiopeptides is a well-described phenomenon [25]. Based on these previous findings, we hypothesize that glomerular filtration of the small size DV1-k-(DV3)-based radioligands (<50 kDa) is followed by a firm interaction with the negatively charged transport proteins of the proximal tubule. This is attributed to the endocytic transporters megalin and cubulin, which are abundantly present in the renal proximal tubule and are involved in protein reabsorption. This interaction could explain the high accumulation of radioactivity in the renal cortex. Co-infusion with Gelofusine^®^ has shown to enhance urinary excretion of megalin transport substrates. Further research will be required to confirm that kidney retention of DV1-k-(DV3)-based radioligands can be reduced by Gelofusine^®^ saturation of renal proximal tubule transporters. In conclusion, we confirmed that the uptake profiles of the diagnostic and therapeutic radioligands are comparable, which is desirable when applying the theranostic approach.

**In vivoradiometabolites.** In vivo radiometabolite analyses were performed for [^18^F]AlF-NOTA-DV1-k-(DV3). Approximately 86 ± 0.1% of radioactivity in plasma of wild-type mice corresponded to the parent radioligand at 15 min p.i. (Figure S27), while in urine 37 ± 2% of the parent radioligand was still intact at 75 min p.i. (Figure S28). As hypothesized, the D-amino acid peptide sequence contributes to a high in vivo plasma stability of [^18^F]AlF-NOTA-DV1-k-(DV3) as the D-conformation peptides are not expected to be substrates for enzymatic degradation.

**Figure 4:**
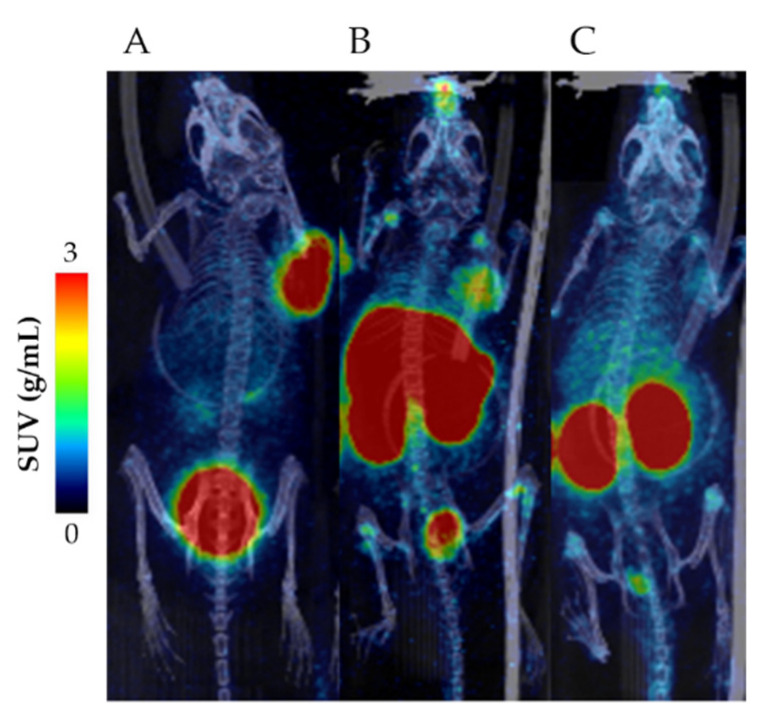
PET/CT imaging in U87.hCXCR4 tumor-bearing mice. MIP image (50–60 min p.i.): (**A**) [^68^Ga]PentixaFor, (**B**) [^18^F]AlF-NOTA-DV1-k-(DV3), (**C**) [^18^F]AlF-NOTA-DV1-k-(DV3) + 5 mg/kg AMD3100.

**Figure 5:**
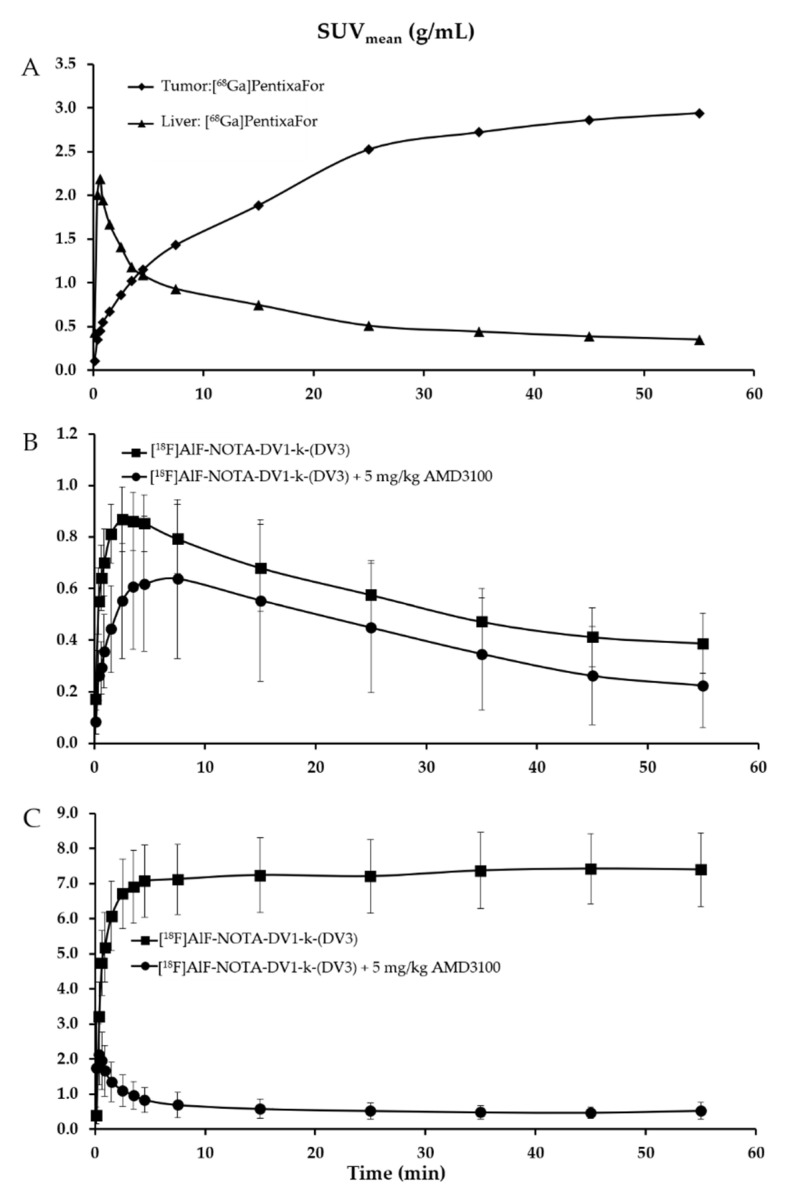
PET/CT imaging in U87.hCXCR4 tumor-bearing mice. (**A**) [^68^Ga]PentixaFor: TACs expressed in SUV_mean_ for hCXCR4-expressing tumors (*n* = 1) and mCXCR4-expressing liver (*n* = 1), [^18^F]AlF-NOTA-DV1-k-(DV3) and [^18^F]AlF-NOTA-DV1-k-(DV3) + 5 mg/kg AMD3100: TACs expressed in SUV_mean_ for hCXCR4-expressing tumors ((**B**), *n* = 4) and mCXCR4-expressing liver ((**C**), *n* = 4).

**Biodistribution of [^18^F]AlF-NOTA-DV1-k-(DV3) in a hCXCR4-expressing tumor model.** Table 4 summarizes the [^18^F]AlF-NOTA-DV1-k-(DV3) uptake in major organs and tumor at 75 min p.i. in U87.hCXCR4 tumor-bearing mice. High accumulation of [^18^F]AlF-NOTA-DV1-k-(DV3) was found in kidney and mCXCR4-expressing organs with CXCR4-specific uptake in liver, spleen and bone, with mean SUV-values of 8.2 ± 1.0, 2.5 ± 1.0 and 0.4 ± 0.1, respectively. Interestingly, [^18^F]AlF-NOTA-DV1-k-(DV3) showed significantly lower tumor uptake (SUV_mean_ 0.6 ± 0.2) than [^68^Ga]PentixaFor (SUV_mean_ 2.9) (Figure 4 and Figure 5). Tumor accumulation of [^18^F]AlF-NOTA-DV1-k-(DV3) with co-injection of AMD3100 was not significantly reduced compared to naïve conditions. However, tumor-to-muscle and tumor-to-blood ratios clearly indicate specific binding of [^18^F]AlF-NOTA-DV1-k-(DV3) to hCXCR4 in vivo, SUV_t/muscle_ 12.4 ± 2.9 (naïve) vs. 4.2 ± 1.1 (* *p* ≤ 0.01, co-injection with AMD3100) and SUV_t/blood_ 4.6 ± 0.9 (naïve) vs. 1.7 ± 0.5 (** *p* ≤ 0.001, co-injection with AMD3100). When comparing tumor uptake kinetics, [^68^Ga]PentixaFor continues to accumulate in the tumor to reach a plateau at the end of the scanning window (60 min). Tumor concentration of [^18^F]AlF-NOTA-DV1-k-(DV3) on the other hand, shows a slow wash-out over time.

The lower tumor accumulation of [^18^F]AlF-NOTA-DV1-k-(DV3) can be partially explained by the affinity of [^18^F]AlF-NOTA-DV1-k-(DV3) toward both mCXCR4 and hCXCR4. Indeed, high CXCR4 expression in the liver [26] combined with the high cardiac output to this organ (±25%) may result in a large fraction of [^18^F]AlF-NOTA-DV1-k-(DV3) binding to mCXCR4 in the liver before it reaches the tumor site, resulting in lower tumor accumulation compared to [^68^Ga]PentixaFor, despite similar in vitro affinity. The same principle accounts for cold competitor, AMD3100, which might contribute to the partial blocking of tumor uptake. This sink effect in the liver was previously observed for other radioligands with high mCXCR4 affinity; however this effect is more pronounced with our radioligand and might be explained by the higher affinity to mCXCR4 of [^18^F]AlF-NOTA-DV1-k-(DV3) compared to the reported radioligands [27]. This aspect must be taken into consideration when comparing the respective biodistribution patterns and tumor-to-background ratios for [^18^F]AlF-NOTA-DV1-k-(DV3) vs. [^68^Ga]PentixaFor, which is selective for hCXCR4.

**Increasing mass injection in a hCXCR4-expressing tumor model.** Based on previously reported results of Nicolas et al., U87.hCXCR4 tumor-bearing mice were co-injected with [^18^F]AlF-NOTA-DV1-k-(DV3) and different mass doses of the vector molecule DV1-k-(DV3) to explore whether an increasing mass of DV1-k-(DV3) could moderately circumvented trapping of [^18^F]AlF-NOTA-DV1-k-(DV3) in the liver by partially blocking mCXCR4 in the liver and therefore increasing the fraction of [^18^F]AlF-NOTA-DV1-k-(DV3) available to bind to other tissues, which might result in an increase of the tumor-to-liver ratio [28].

Accumulation in the liver was reduced to mean SUV-values of 1.2 ± 0.1, 1.2 ± 0.4 and 0.7 ± 0.1 whereas uptake of [^18^F]AlF-NOTA-DV1-k-(DV3) at the tumor site increased to mean SUV-values of 0.7 ± 0.1, 0.9 ± 0.3 and 0.6 ± 0.2 for injected masses of DV1-k-(DV3) of 7, 14 and 24 nmol respectively (Table 5 and Table S3). The highest tumor-to liver ratio (SUV_mean t/liver_ 0.9 ± 0.2) was observed for an injected mass of 24 nmol, which is significantly higher than the control condition without co-injection of cold peptide (SUV_mean t/liver_ 0.1 ± 0.02). However, tumor-to-muscle ratios are higher for the naïve condition (SUV_mean t/muscle_ 12.4 ± 2.9) compared to the increased peptide mass conditions (SUV_mean t/muscle_ 5.4 ± 2.2, 4.8 ± 2.1 and 3.0 ± 0.4 for 7, 14 and 24 nmol respectively) because of the higher blood and muscle concentration of the radioligand. A different administration route for the cold CXCR4 competitor to only saturate liver binding sites or an animal model with CXCR4 expression more relevant to humans would improve our understanding of the in vivo behavior of these radioligands.

**Figure 6:**
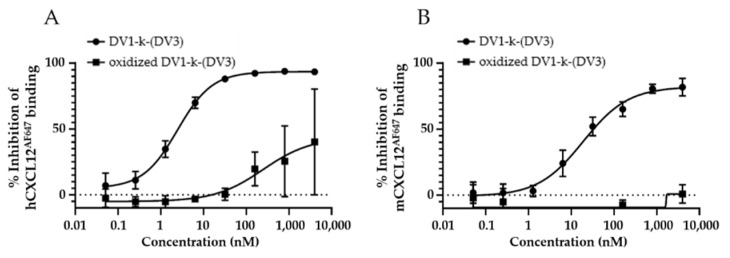
Binding affinities of DV1-k-(DV3) and oxidized DV1-k-(DV3) as reference to hCXCR4 (**A**) and mCXCR4 (**B**). Affinities for hCXCR4 were determined using Jurkat cells and human CXCL12^AF647^ as fluorescent ligand. Affinities for mCXCR4 were determined using U87.mCXCR4 and murine CXCL12^AF647^ as fluorescent ligand. All data are expressed as mean ± SD (*n* = 3).

**In vivo oxidation of DV1-k-(DV3).** As shown above, [^68^Ga]PentixaFor accumulation at the tumor site increased over time and reached a plateau at the end of the scanning window. In contrast, tumor concentration of [^18^F]AlF-NOTA-DV1-k-(DV3) shows a slow wash-out over time. This effect might be explained by the presence of two cysteines in the DV1 sequence that could be sensitive to oxidation in vivo. It is clear from the in vitro data in U87.hCXCR4 cells that [^18^F]AlF-NOTA-DV1-k-(DV3) displays specific binding to hCXCR4. In vivo oxidation of one or both cysteines can however have a profound impact on the CXCR4 affinity. To confirm this hypothesis, oxidized (S-S) DV1-k-(DV3) was also evaluated for affinity toward hCXCR4 and mCXCR4 (Figure 6). The intramolecular S-S bond leads indeed to loss of affinity for both hCXCR4 and mCXCR4, and might explain partially the slow wash-out over time in tumor tissue. Derivatives of DV1-k-(DV3) where cysteines are substituted with other amino acids are already synthesized and are currently under evaluation for their binding affinity and tumor retention.

## 4. Conclusions

DV1-k-(DV3) was successfully derivatized allowing labeling with different radionuclides, for both diagnostic and therapeutic purposes. All DV1-k-(DV3)-based ligands displayed high in vitro binding affinity for hCXCR4 and mCXCR4. [^1^^8^F]AlF-NOTA-DV1-k-(DV3) was produced in an automated synthesis module using a cGMP-compatible Al^18^F-labeling protocol, facilitating future clinical implementation. The D-amino acid-based peptide of DV1-k-(DV3) resulted in high in vitro and in vivo stability of the corresponding radioligands. Furthermore, the diagnostic radiotracers, [^18^F]AlF-NOTA-DV1-k-(DV3) and [^68^Ga]Ga-DOTA-DV1-k-(DV3), and their therapeutic companion [^177^Lu]Lu-DOTA-DV1-k-(DV3), exhibit the same favorable pharmacokinetic profile in vivo, showing the potential of DV1-k-(DV3) for theranostic applications. In vivo specific binding of [^18^F]AlF-NOTA-DV1-k-(DV3) to hCXCR4, based on tumor-to-muscle and tumor-to-blood ratios, and mCXCR4 was confirmed in hCXCR4-expressing tumor-bearing mice. Although the in vitro binding affinity to hCXCR4 of DV1-k-(DV3) derivatives and [^nat^Ga]PentixaFor was similar, lower tumor uptake for [^18^F]AlF-NOTA-DV1-k-(DV3) was observed. High binding affinity to mCXCR4 of the DV1-k-(DV3) scaffold and high expression of CXCR4 in mouse liver make evaluation of the clinical potential of this new class of CXCR4-targeting radiotracers in tumor mouse models difficult. Nonetheless, [^18^F]AlF-NOTA-DV1-k-(DV3) has proven its potential as translational diagnostic radiotracer for imaging of CXCR4 up-regulation, not only in an oncological setting but potentially also in preclinical models of myocardial infarction, infection or inflammation. As CXCR4 is not expressed in healthy human liver, the findings in mice are not predictive for the potential clinical performance of this novel class of CXCR4-targeting radiotracers.

## Figures and Tables

**Table 1:** Binding affinities (half-maximal inhibitory concentration (IC_50_) in nM) of [^nat^Ga]PentixaFor, AMD3100 and DV1-k-(DV3) derivatives for human CXCR4 (hCXCR4). Affinities for hCXCR4 were determined using Jurkat cells and human CXCL12^AF647^. All data are expressed as mean ± SD (*n* = 3).

Peptide	IC_50_ [nM] to hCXCR4
[^nat^Ga]PentixaFor	8.6 ± 1.1
AMD3100	40.4 ± 3.5
DV1	241.7 ± 18.1
DV3	>1000
DV1-k-(DV3)	2.9 ± 0.4
NOTA-DV1-k-(DV3)	14.2 ± 1.4
DOTA-DV1-k-(DV3)	27.3 ± 2.8
AlF-NOTA-DV1-k-(DV3)	5.3 ± 0.9
[^nat^Ga]Ga-DOTA-DV1-k-(DV3)	4.3 ± 0.3
[^nat^Lu]Lu-DOTA-DV1-k-(DV3)	2.9 ± 0.6
[^nat^Bi]Bi-DOTA-DV1-k-(DV3)	8.8 ± 1.1
La-DOTA-DV1-k-(DV3)	14.7 ± 2.1

**Table 2:** Binding affinities (IC_50_ in nM) of [^nat^Ga]PentixaFor, AMD3100, DV1-k-(DV3) and AlF-NOTA-DV1-k-(DV3) to murine CXCR4 (mCXCR4). Affinities to mCXCR4 were determined using U87.mCXCR4 and murine CXCL12^AF647^ as fluorescent ligand. All data are expressed as mean ± SD (*n* = 3).

**Peptide**	**IC_50_ [nM] to mCXCR4**
[^nat^Ga]PentixaFor	>1000
AMD3100	123.4 ± 17.0
DV1-k-(DV3)	41.5 ± 12.3
AlF-NOTA-DV1-k-(DV3)	33.4 ± 13.5

**Table 3:** Total cell-bound fraction (membrane-bound + internalized fraction) of the respective radioligands and the percentage of internalized radioligand were determined using hCXCR4-expressing U87 cells (2.5 × 10^5^ cells/well, 60 min at 37 °C) in absence or presence of AMD3100 (100 µM). Statistical analysis independent-samples *t*-test. All data are expressed as mean ± SD (*n* = 3). * *p* ≤ 0.01, ** *p* ≤ 0.001.

	Total Cell-Bound Fraction [% of Applied Activity]		Internalized Fraction [% of Total Cell-Bound Fraction]
**AMD3100**		**100 µM**	
[^68^Ga]PentixaFor	8.9 ± 0.3	0.1 ± 0.003 **	10.0 ± 0.1
[^18^F]AlF-NOTA-DV1-k-(DV3)	6.0 ± 0.1	1.1 ± 0.1 **	20.1 ± 2.0
[^68^Ga]Ga-DOTA-DV1-k-(DV3)	6.4 ± 0.1	1.9 ± 0.2 **	31.2 ± 2.4
[^177^Lu]Lu-DOTA-DV1-k-(DV3)	4.6 ± 0.5	0.5 ± 0.04 *	29.4 ± 0.8

**Table 4:** Ex vivo biodistribution in U87.hCXCR4 tumor-bearing mice 75 min p.i. (expressed in SUV). Statistical analysis independent-samples *t*-test. All data are expressed as mean ± SD (*n* = 4). * *p* ≤ 0.01, ** *p* ≤ 0.001.

	[^18^F]AlF-NOTA-DV1-k-(DV3)	[^18^F]AlF-NOTA-DV1-k-(DV3) + 5 mg/kg AMD3100
liver	8.2 ± 1.0	0.9 ± 0.3 **
spleen	2.5 ± 1.0	0.5 ± 0.1 *
bone	0.4 ± 0.1	0.1 ± 0.04 *
tumor	0.6 ± 0.2	0.4 ± 0.2
muscle	0.05 ± 0.02	0.1 ± 0.1
blood	0.1 ± 0.1	0.3 ± 0.2
t/muscle	12.4 ± 2.9	4.2 ± 1.1 *
t/blood	4.6 ± 0.9	1.7 ± 0.5 **

**Table 5:** Increased mass of DV1-k-(DV3). Ex vivo biodistribution in U87.hCXCR4 tumor-bearing mice 75 min p.i. (expressed in SUV). Statistical analysis independent-samples *t*-test. All data are expressed as mean ± SD. * *p* < 0.05, ** *p* ≤ 0.01, *** *p* ≤ 0.001.

	[^18^F]AlF-NOTA-DV1-k-(DV3)				
AMD3100	0 nmol *n* = 4				157 nmol *n* = 4
DV1-k-(DV3)		7 nmol *n* = 3	14 nmol *n* = 4	24 nmol *n* = 4	
liver	8.2 ± 1.0	1.2 ± 0.1 ***	1.2 ± 0.4 ***	0.7 ± 0.1 ***	0.9 ± 0.3 ***
spleen	2.5 ± 1.0	0.5 ± 0.1 **	0.6 ± 0.2 *	0.4 ± 0.1 **	0.5 ± 0.1 **
bone	0.4 ± 0.1	0.2 ± 0.04 **	0.3 ± 0.1 *	0.2 ± 0.04 **	0.1 ± 0.04 **
tumor	0.6 ± 0.2	0.7 ± 0.1	0.9 ± 0.3	0.6 ± 0.2	0.4 ± 0.2
t/liver	0.1 ± 0.02	0.6 ± 0.1 **	0.8 ± 0.2 **	0.9 ± 0.2 ***	0.5 ± 0.2 *
t/muscle	12.4 ± 2.9	5.4 ± 2.2 **	4.8 ± 2.1 **	3.0 ± 0.4 **	4.2 ± 1.1 **

## Data Availability

Not applicable.

## Supplementary Materials

The following are available online at https://www.mdpi.com/article/10.3390/pharmaceutics13101619/s1, Figure S1: Structure of resin-bound DV1-k-(DV3), Table S1: Gradient used for the HPLC purification of NOTA-DV1-k-(DV3) and DOTA-DV1-k-(DV3), Figure S2: HRMS chromatogram of NOTA-DV1-k-(DV3), Figure S3: HPLC chromatogram of NOTA-DV1-k-(DV3) (UV-220 nm), Figure S4: HRMS chromatogram of DOTA-DV1-k-(DV3), Figure S5: HPLC chromatogram of DOTA-DV1-k-(DV3) (UV-220 nm), Figure S6: HRMS chromatogram of AlF-NOTA-DV1-k-(DV3), Figure S7: HPLC chromatogram of AlF-NOTA-DV1-k-(DV3) (UV-220 nm), Figure S8: HRMS chromatogram of [^nat^Ga]Ga-DOTA-DV1-k-(DV3), Figure S9: HPLC chromatogram of [^nat^Ga]Ga-DOTA-DV1-k-(DV3) (UV-220 nm), Figure S10: HRMS chromatogram of [^nat^Lu]Lu-DOTA-DV1-k-(DV3), Figure S11: HPLC chromatogram of [^nat^Lu]Lu-DOTA-DV1-k-(DV3) (UV-220 nm), Figure S12: HRMS chromatogram of [^nat^Bi]Bi-DOTA-DV1-k-(DV3), Figure S13: HPLC chromatogram of [^nat^Bi]Bi-DOTA-DV1-k-(DV3) (UV-220 nm), Figure S14: HRMS chromatogram of La-DOTA-DV1-k-(DV3), Figure S15: HPLC chromatogram of La-DOTA-DV1-k-(DV3) (UV-220 nm), Figure S16: HRMS chromatogram of oxidized DV1-k-(DV3), Figure S17: HRMS chromatogram of [^nat^Ga]PentixaFor, Figure S18: HPLC chromatogram of [^nat^Ga]PentixaFor (UV-254 nm), Figure S19: RadioHPLC chromatogram of [^18^F]AlF-NOTA-DV1-k-(DV3), Figure S20: RadioHPLC chromatogram of [^68^Ga]Ga-DOTA-DV1-k-(DV3), Figure S21: RadioHPLC chromatogram of [^68^Ga]PentixaFor, Figure S22: RadioHPLC chromatogram of [^177^Lu]Lu-DOTA-DV1-k-(DV3), Figure S23: In vitro stability study of [^18^F]AlF-NOTA-DV1-k-(DV3) in formulation buffer (A, room temperature, 5 h) and human serum (B, 37 °C, 2 h), Figure S24: In vitro stability study (2 h) of [^68^Ga]Ga-DOTA-DV1-k-(DV3) in formulation buffer (A, room temperature) and human serum (B, 37 °C), Figure S25: In vitro stability study (9 days) of [^177^Lu]Lu-DOTA-DV1-k-(DV3) in formulation buffer (A, 4 °C) and human serum (B, 37 °C), Table S2: Ex vivo biodistribution in healthy mice 75 min p.i. (expressed in SUV). [^68^Ga]PentixaFor (*n* = 3), [^68^Ga]PentixaFor + 5 mg/kg AMD3100 (*n* = 1), [^18^F]AlF-NOTA-DV1-k-(DV3) (*n* = 5), [^18^F]AlF-NOTA-DV1-k-(DV3) + 5 mg/kg AMD3100 (*n* = 2), [^68^Ga]Ga-DOTA-DV1-k-(DV3) (*n* = 4), [^68^Ga]Ga-DOTA-DV1-k-(DV3) + 5 mg/kg AMD3100 (*n* = 4), [^177^Lu]Lu-DOTA-DV1-k-(DV3) (*n* = 3) and [^177^Lu]Lu-DOTA-DV1-k-(DV3) + 5 mg/kg AMD3100 (*n* = 3). Statistical analysis independent-samples t-test. * *p* < 0.05, ** *p* ≤ 0.01, *** *p* ≤ 0.001, Figure S26: Ex vivo autoradiography of kidney, liver and spleen of healthy mice 75 min p.i. of [^18^F]AlF-NOTA-DV1-k-(DV3), [^18^F]AlF-NOTA-DV1-k-(DV3) + 5 mg/kg AMD3100, [^68^Ga]Ga-DOTA-DV1-k-(DV3) and [^68^Ga]Ga-DOTA-DV1-k-(DV3) + 5 mg/kg AMD3100, Figure S27: In vivo radiometabolite study of [^18^F]AlF-NOTA-DV1-k-(DV3) in plasma of healthy mice 15 min p.i., Figure S28: In vivo radiometabolite study of [^18^F]AlF-NOTA-DV1-k-(DV3) in urine of healthy mice 75 min p.i., Table S3: Ex vivo biodistribution in U87.hCXCR4 tumor-bearing mice 75 min p.i. (expressed in SUV). Statistical analysis independent-samples *t*-test. * *p* < 0.05, ** *p* ≤ 0.01, *** *p* ≤ 0.001. References [16,17] are cited in the Supplementary Materials.
