# Supplementary material for: D-Peptide-Based Probe for CXCR4-Targeted Molecular Imaging and Radionuclide Therapy
Source: Pharmaceutics. 2021 Oct 5;13(10):1619. doi: 10.3390/pharmaceutics13101619 (PMC8537445; doi:10.3390/pharmaceutics13101619)
Supplement: Supplementary file 1 [file pharmaceutics-13-01619-s001.zip › pharmaceutics-1360664-supplementary.pdf]

pharmaceutics

MDPI

D-Peptide-Based Probe for CXCR4-Targeted Molecular Imaging and Radionuclide Therapy

Kaat Luyten, Tom Van Loy, Christopher Cawthorne, Christophe M. Deroose, Dominique Schols, Guy Bormans and Frederik Cleeren

1. Synthesis

1.1. NOTA-DV1-k-(DV3) and DOTA-DV1-k-(DV3)

DV1-K-(DV3) resin (Pepmic Co., Ltd (Suzhou, China), 100 mg, 0.02 mmol, 1 eq. (Figure S1)) was allowed to swell in dichloromethane (DCM) and the resin was washed two times with 2 mL of dimethylformamide (DMF). 2-(7-Aza-1H-benzotriazole-1-yl)-1,1,3,3-tetramethyluronium hexafluorophosphate (HATU, 0.1 mmol, 5 eq.), N-Ethyldiisopropylamine (DIPEA, 0.2 mmol, 10 eq.) and 2-(4,7-bis(2-(tert-butoxy)-2-oxoethyl)-1,4,7-triazonan-1-yl)acetic acid (NOTA(tBu)$_2$, 0.05 mmol, 2.5 eq., CheMatech, Dijon, France) or 2-(4,7,10-tris(2-(tert-butoxy)-2-oxoethyl)-1,4,7,10-tetraazacyclododecan-1-yl)acetic acid (DOTA(tBu)$_3$, (0.05 mmol, 2.5 eq., CheMatech , Dijon, France) were added in DMF. The mixture was allowed to react for 2 h at room temperature. Next, the resin was washed three times with 2 mL DMF and three times with 2 mL DCM. The peptides were cleaved from the resin using a mixture of trifluoroacetic acid, water and tri-isopropyl silane (92.5; 2.5; 5% *v*/*v*). Cleaved NOTA-DV1-k-(DV3) or DOTA-DV1-k-(DV3) was dissolved in 1 mL acetonitrile/water (50;50) and purified using HPLC at 220 nm. A gradient (Table S1) of water (0.1% trifluoroacetic acid (TFA)) with acetonitrile (0.1% TFA) on a Waters XBridge C$_{18}$ prep column (10 × 250 mm; 5 µm) at a flow rate of 5 mL/min was used. Both compounds eluted with a retention time of 10.3 min. Fractions were collected, concentrated under vacuum, and cooled in dry ice for 10 min followed by lyophilization overnight and stored at −20 °C. LC-HRMS analysis was performed.

NOTA-DV1-k-(DV3): $C_{178}H_{277}N_{55}O_{46}S_2$ theoretical neutral average mass (electrospray ionization (ESI)-LCMS) 3987.58 Da; found 3987.53 ± 0.02 Da. DOTA-DV1-k-(DV3): $C_{182}H_{284}N_{56}O_{48}S_2$ theoretical neutral average mass (ESI-LCMS) 4088.68 Da; found 4088.57 ± 0.01 Da.

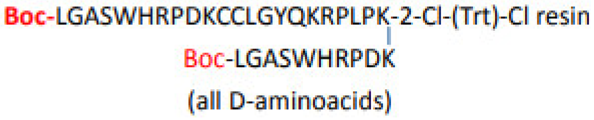

Figure S1. Structure of resin-bound DV1-k-(DV3).

Table S1. Gradient used for the HPLC purification of NOTA-DV1-k-(DV3) and DOTA-DV1-k-(DV3).

Time	Water (0.1% TFA)	Acetonitrile (0.1% TFA)
0 min	83%	17%
15 min	70%	30%
15–20 min	20%	80%
20–30 min	83%	17%

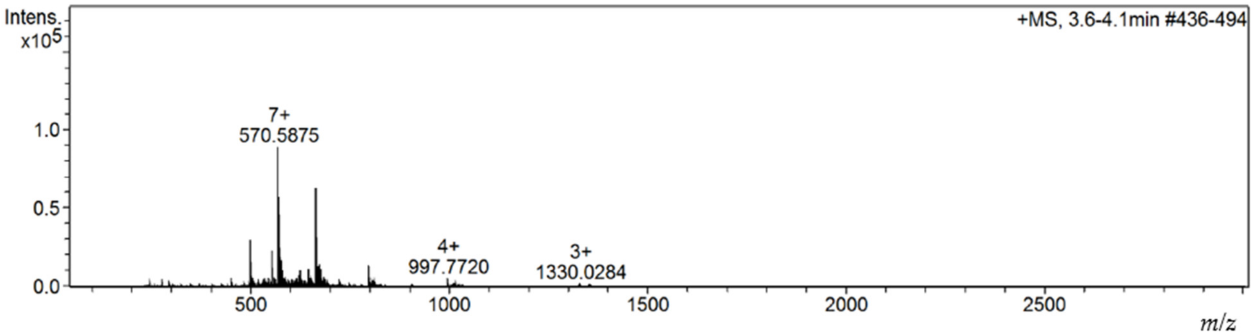

Figure S2. HRMS chromatogram of NOTA-DV1-k-(DV3).

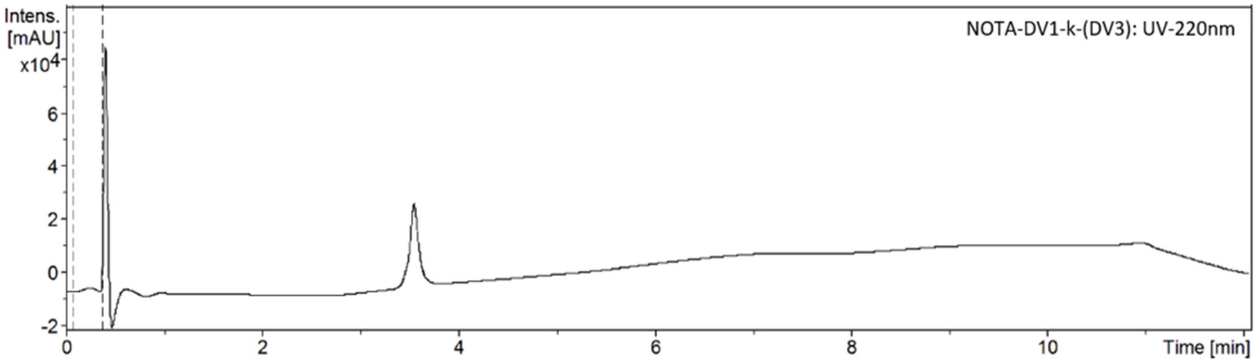

Figure S3. HPLC chromatogram of NOTA-DV1-k-(DV3) (UV-220nm).

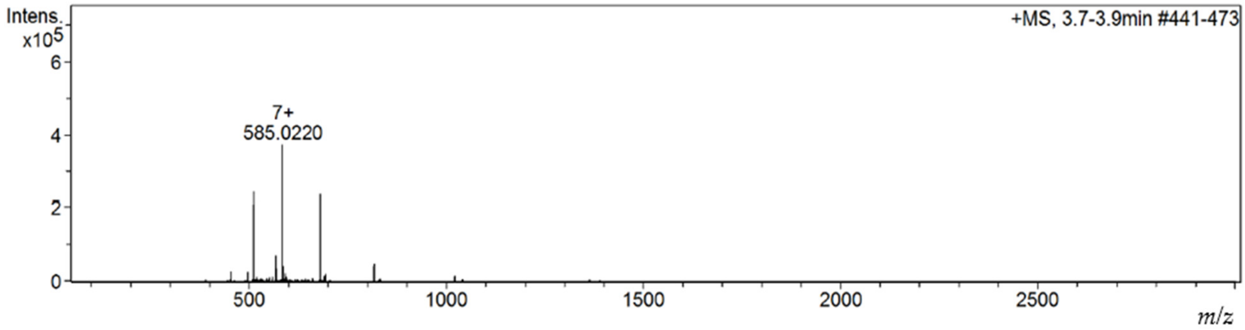

Figure S4. HRMS chromatogram of DOTA-DV1-k-(DV3).

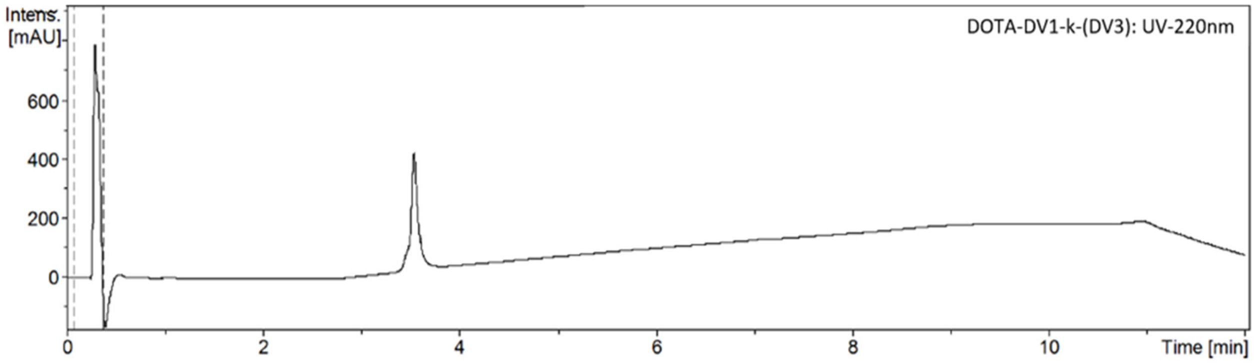

Figure S5. HPLC chromatogram of DOTA-DV1-k-(DV3) (UV-220nm).

1.2. AlF-NOTA-DV1-k-(DV3)

A solution containing $AlCl_3$ (490 µL, 5.1 mM in 0.1 M sodium acetate, pH 4.1, 10 eq.) and NaF (10 µL, 10 mg/mL in 0.1 M sodium acetate, pH 4.1, 10 eq.) was allowed to stir for 5 min at room temperature. After addition of 500 µL absolute ethanol (EtOH), the solution was added to the NOTA-DV1-k-(DV3) (1.0 mg, 1 eq.) (Mr 3987.5 g/mol) and vortexed. The reaction mixture was allowed to react for 30 min at 95 °C. Metal-free water (20 mL, HPCE grade water (Sigma Aldrich, Saint Louis, MO, USA) was used to dilute the reaction mixture after cooling for 10 min and this was loaded onto an activated Sep-Pak Plus Light C_{18}. The vial was rinsed with 2 mL metal-free water and this was also loaded onto the activated Sep-Pak Plus Light C_{18}. The peptide was eluted with 0.5 mL acetonitrile and 1 mL of metal-free water was added. The mixture was sonicated for 5 min and filtered using a Captiva PTFE + GF 0.45 µm filter from Agilent. The mixture was subsequently cooled in dry ice for 10 min followed by lyophilization overnight and stored at −20 °C. $C_{178}H_{275}N_{55}O_{46}S_2AlF$ theoretical neutral average mass (ESI-LCMS) 4031.55 Da; found 4031.48 ± 0.02 Da.

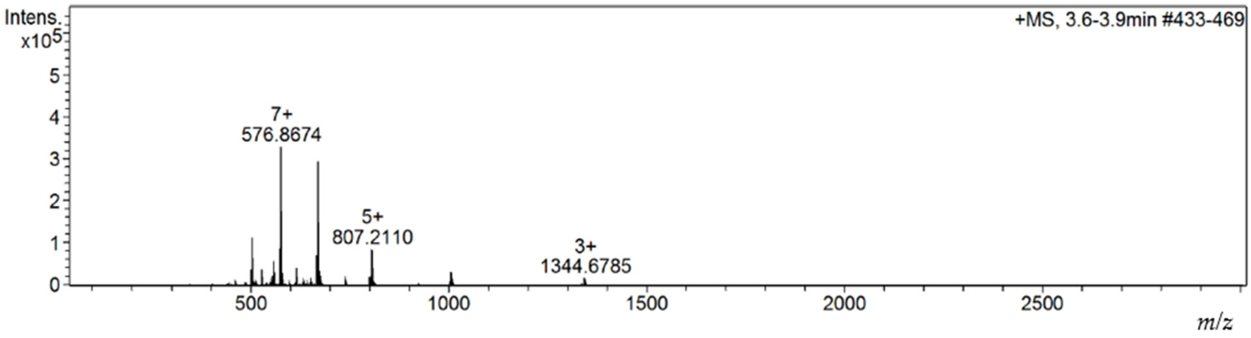

Figure S6. HRMS chromatogram of AlF-NOTA-DV1-k-(DV3).

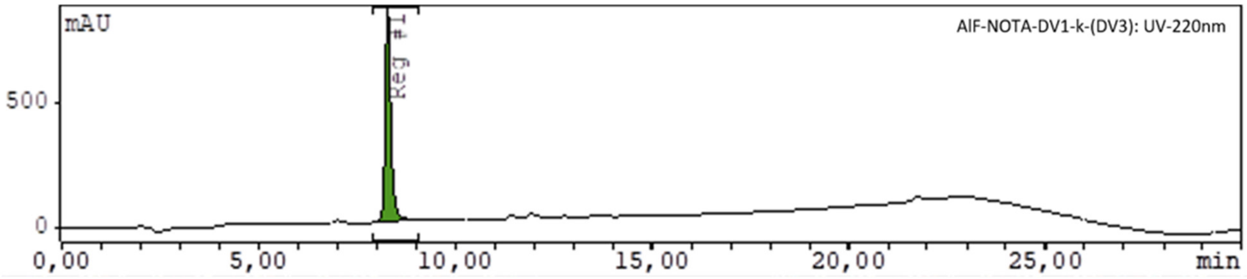

Figure S7. HPLC chromatogram of AlF-NOTA-DV1-k-(DV3) (UV-220nm).

1.3. [natGa]Ga-DOTA-DV1-k-(DV3)

The protocol for the $^{nat}Ga^{3+}$-labelling of DOTA-DV1-k-(DV3) was based on the protocol in the paper of Suzuki et al. [1]. A solution was made containing $Ga(NO_3)_3$ (220 µL, 20 mM in 0.1 M sodium acetate, pH 4.1, 20 eq.) and the DOTA-bound peptide (0.9 mg, 1 mM, 1 eq.) (Mr 4088.69 g/mol). The reaction mixture was allowed to react for 30 min at 95 °C. After cooling, the mixture was diluted with 2 mL metal-free water and this was loaded onto the activated Sep-Pak Plus Light C_{18}. The vial was rinsed with 1 mL metal-free water and this was also loaded onto the activated Sep-Pak Plus Light C_{18}. The peptide was eluted with 1 mL EtOH into a vial. EtOH was evaporated under vacuum and 1 mL of metal-free water and 500 µL of acetonitrile (LC-HRMS grade) was added. The mixture was sonicated for 5 min and filtered using a Captiva PTFE + GF 0.45 µm filter from Agilent. The mixture was subsequently cooled in dry ice for 10 min followed by lyophilization overnight and stored at −20 °C. $C_{182}H_{282}N_{56}O_{48}S_2Ga$ theoretical neutral average mass (ESI-LCMS) 4156.39 Da; found 4154.80 ± 0.02 Da.

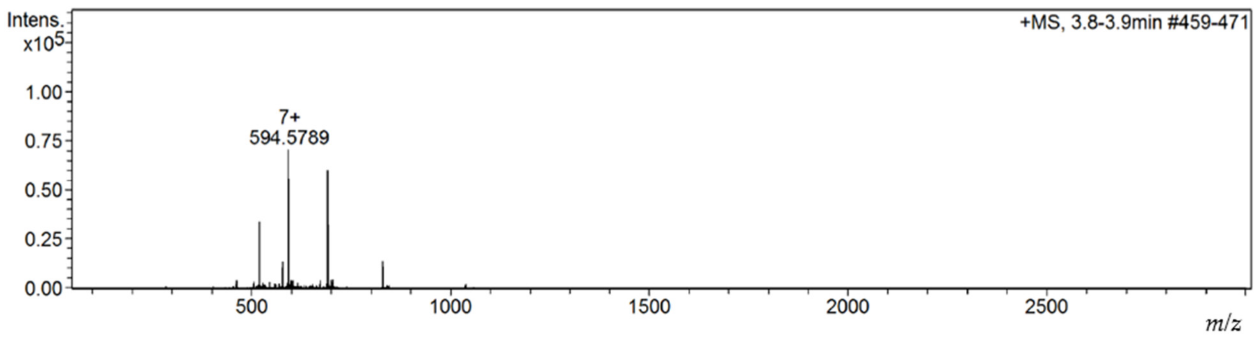

Figure S8. HRMS chromatogram of [natGa]Ga-DOTA-DV1-k-(DV3).

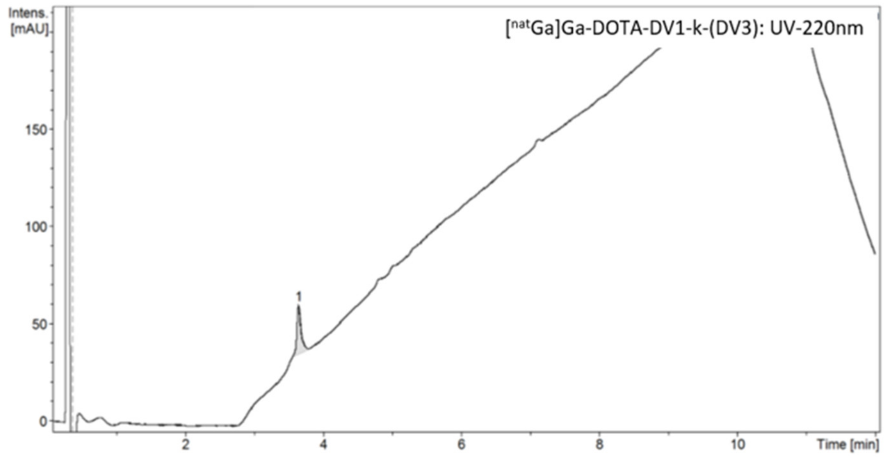

Figure S9. HPLC chromatogram of [natGa]Ga-DOTA-DV1-k-(DV3) (UV-220nm).

1.4. [natLu]Lu-DOTA-DV1-k-(DV3)

The protocol for the $^{nat}Lu^{3+}$-labelling of DOTA-DV1-k-(DV3) was based on the protocol in the paper of Suzuki et al. [1]. A solution was made containing $LuCl_3$ (220 µL, 20 mM in 0.01 M HCl, 20 eq.) and the DOTA-bound peptide (0.9 mg, 1 mM, 1 eq.) (Mr 4088.69 g/mol). The reaction mixture was allowed to react for 30 min at 95 °C. After cooling, the mixture was diluted with 2 mL metal-free water and loaded onto an activated Sep-Pak Plus Light C_{18}. The vial was rinsed with 1 mL metal-free water and this was loaded onto the activated Sep-Pak Plus Light C_{18} as well. The peptide was eluted with 1 mL EtOH into a vial. EtOH was evaporated under vacuum and 1 mL of metal-free water and 500 µL of acetonitrile (LC-HRMS grade) was added. The mixture was sonicated for 5 min and filtered using a Captiva PTFE + GF 0.45 µm filter from Agilent. The mixture was subsequently cooled in dry ice for 10 min followed by lyophilization overnight and stored at −20 °C. $C_{182}H_{281}N_{56}O_{48}S_2Lu$ theoretical neutral average mass (ESI-LCMS) 4260.63 Da; found 4259.14 ± 0.01 Da.

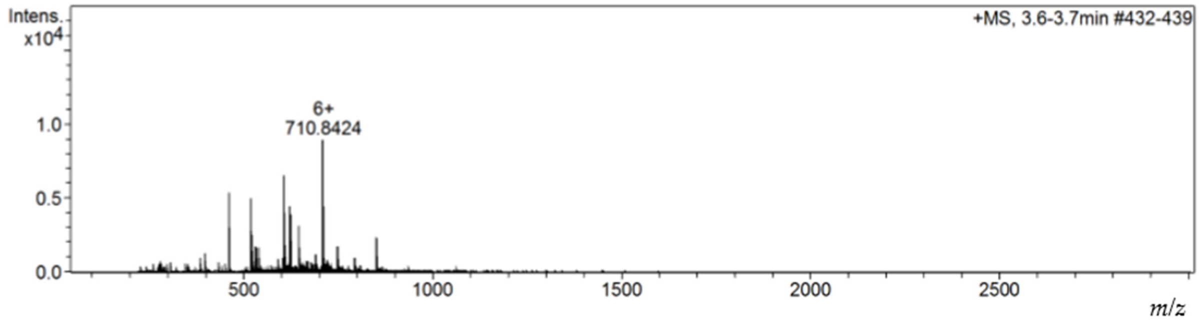

Figure S10. HRMS chromatogram of [natLu]Lu-DOTA-DV1-k-(DV3).

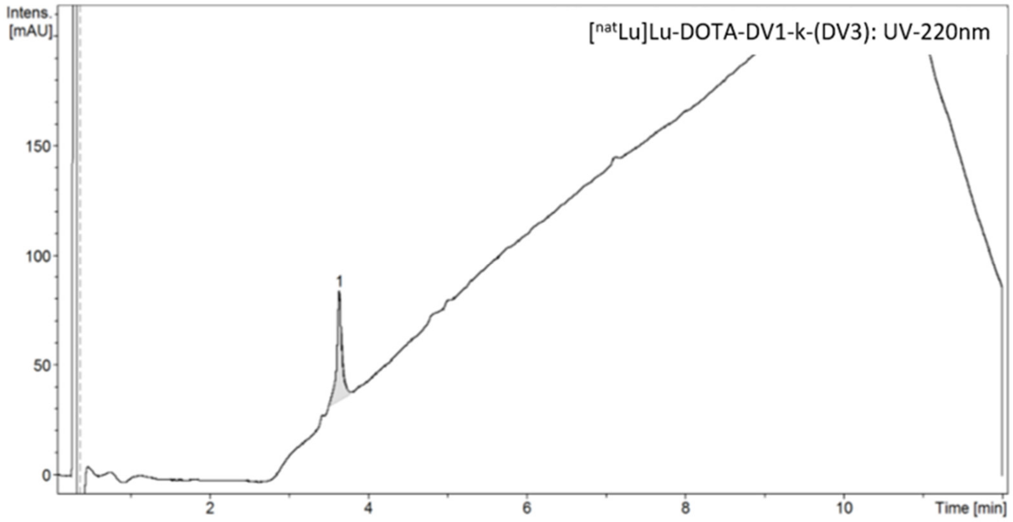

Figure S11. HPLC chromatogram of [natLu]Lu-DOTA-DV1-k-(DV3) (UV-220nm).

1.5. [natBi]Bi-DOTA-DV1-k-(DV3)

The protocol for $^{nat}Bi^{3+}$-labelling of DOTA-DV1-k-(DV3) was adapted from Suzuki et al. [1]. A solution containing $BiCl_3$ (2 mM in 1.2 M sodium acetate, pH 5, 5 eq.) and the DOTA-bound peptide (0.8 mg, 0.4 mM, 1 eq.) (Mr 4088.69 g/mol) in a total volume of 500 µL was prepared. The reaction mixture was allowed to react for 30 min at 95 °C. Metal-free water (2 mL) was used to dilute the reaction mixture after cooling for 10 min and this was loaded onto an activated Sep-Pak Plus Light C_{18}. The vial was rinsed with 2 mL metal-free water and this was also loaded onto the activated Sep-Pak Plus Light C_{18}. The peptide was eluted with 1 mL EtOH into a vial. EtOH was evaporated under vacuum and 1 mL of metal-free water and 500 µL of acetonitrile (LC-HRMS grade) was added. The mixture was sonicated for 5 min and filtered using a Captiva PTFE + GF 0.45 µm filter from Agilent. The mixture was subsequently cooled in dry ice for 10 min followed by lyophilization overnight and later stored at −20 °C. $C_{182}H_{281}N_{56}O_{48}S_2Bi$ theoretical neutral average mass (ESI-LCMS) 4294.64 Da; found 4294.41 ± 0.02 Da.

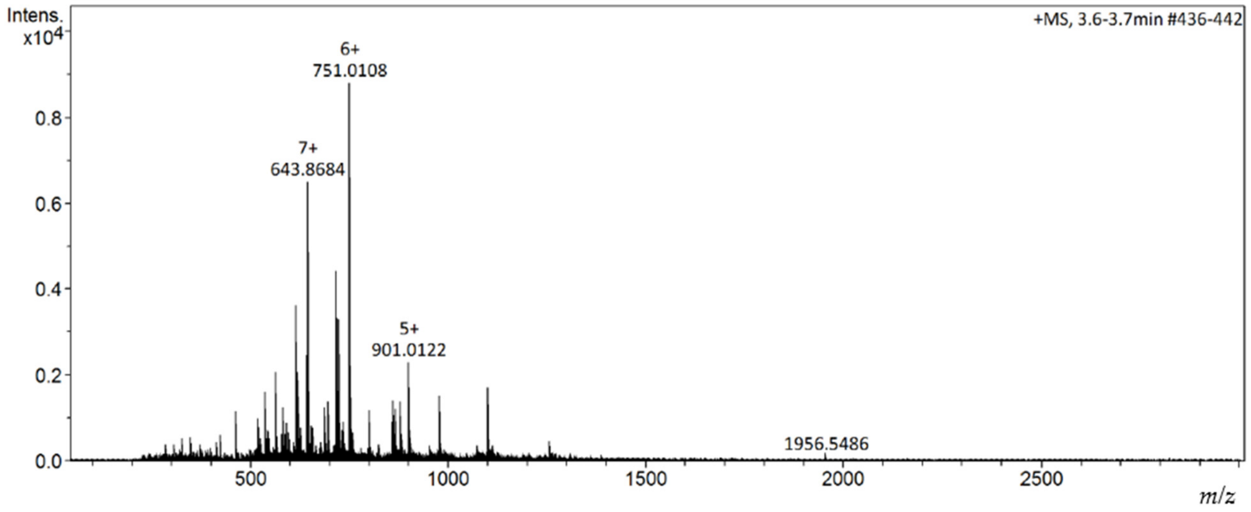

Figure S12. HRMS chromatogram of [natBi]Bi-DOTA-DV1-k-(DV3).

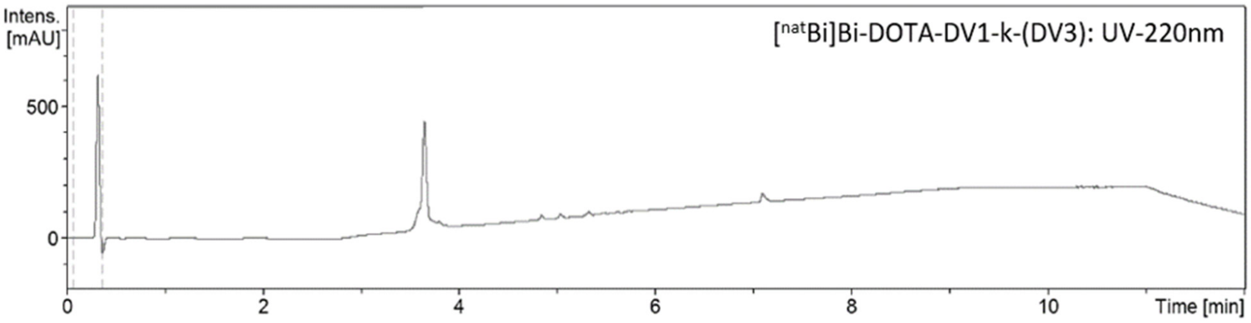

Figure S13. HPLC chromatogram of [natBi]Bi-DOTA-DV1-k-(DV3) (UV-220nm).

1.6. La-DOTA-DV1-k-(DV3)

The protocol for $^{nat}La^{3+}$-labelling of DOTA-DV1-k-(DV3) was adapted from Suzuki et al. [1]. A solution containing $LaCl_3$ (2.5 mM in 1.2 M sodium acetate, pH 5, 5 eq.) and the DOTA-bound peptide (1.09 mg, 0.52 mM, 1 eq.) (Mr 4088.69 g/mol) in a total volume of 500 µL was prepared. The reaction mixture was allowed to react for 30 min at 95 °C. Metal-free water (2 mL) was used to dilute the reaction mixture after cooling for 10 min and this was loaded onto an activated Sep-Pak Plus Light C_{18}. The vial was rinsed with 2 mL metal-free water and this was also loaded onto the activated Sep-Pak Plus Light C_{18}. The peptide was eluted with 1 mL EtOH into a vial. EtOH was evaporated under vacuum and 1 mL of metal-free water and 500 µL of acetonitrile (LC-HRMS grade) was added. The mixture was sonicated for 5min and filtered using a Captiva PTFE + GF 0.45 µm filter from Agilent. The mixture was subsequently cooled in dry ice for 10 min followed by lyophilization overnight and stored at −20 °C. $C_{182}H_{281}N_{56}O_{48}S_2La$ theoretical neutral average mass (ESI-LCMS) 4224.57 Da; found 4224.53 ± 0.03 Da.

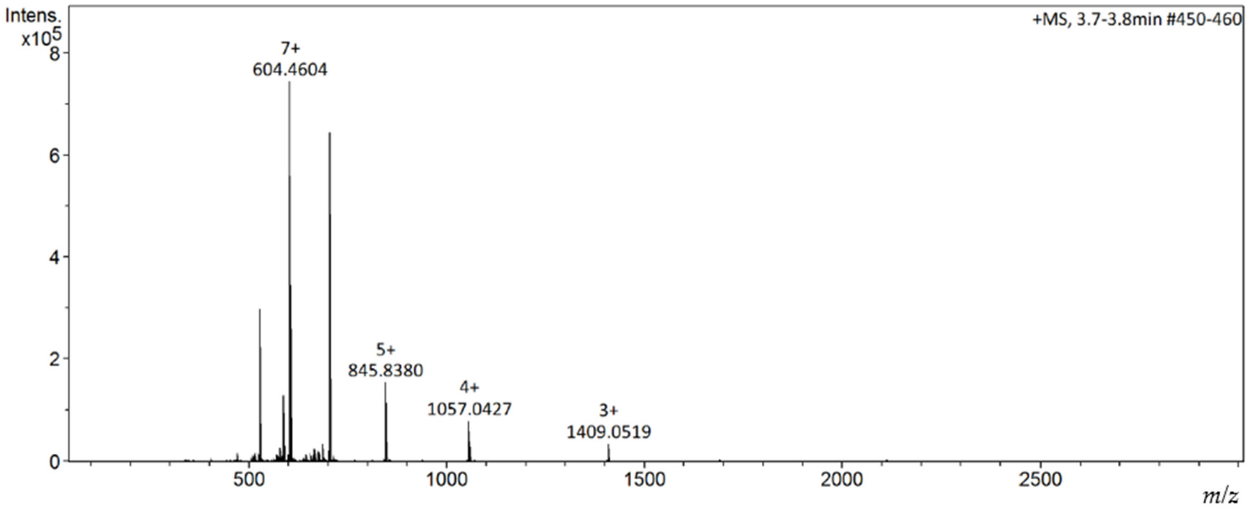

Figure S14. HRMS chromatogram of La-DOTA-DV1-k-(DV3).

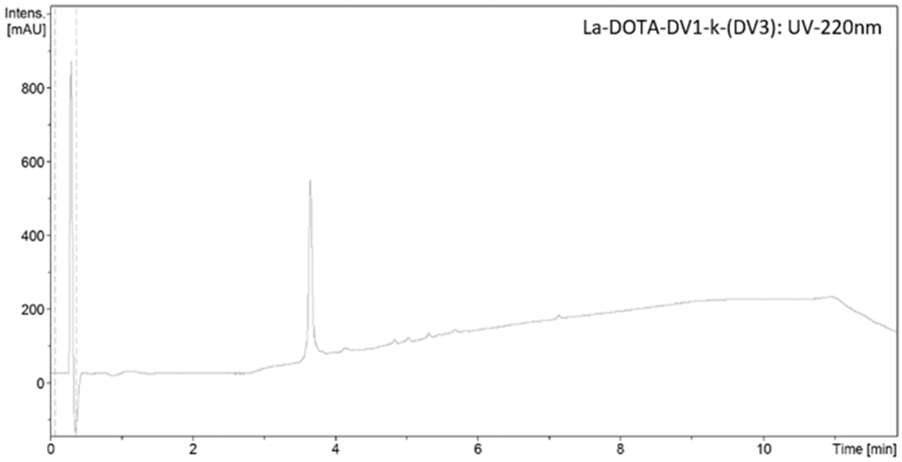

Figure S15. HPLC chromatogram of La-DOTA-DV1-k-(DV3) (UV-220nm).

1.7. Oxidized DV1-k-(DV3)

A solution of 20 µM DV1-k-(DV3) in metal-free water and 20% DMSO was prepared and reacted for 36 h at room temperature. Complete oxidation of both cysteines in the DV1 sequence was confirmed by LC-HRMS analysis. DV1-k-(DV3): $C_{166}H_{258}N_{52}O_{41}S_2$ theoretical neutral average mass (ESI-LCMS) 3702.28 Da; found 3700.34 ± 0.02 Da. Oxidized DV1-k-(DV3): $C_{166}H_{256}N_{52}O_{41}S_2$ theoretical neutral average mass (ESI-LCMS) 3700.27 Da; found 3700.27 ± 0.01 Da.

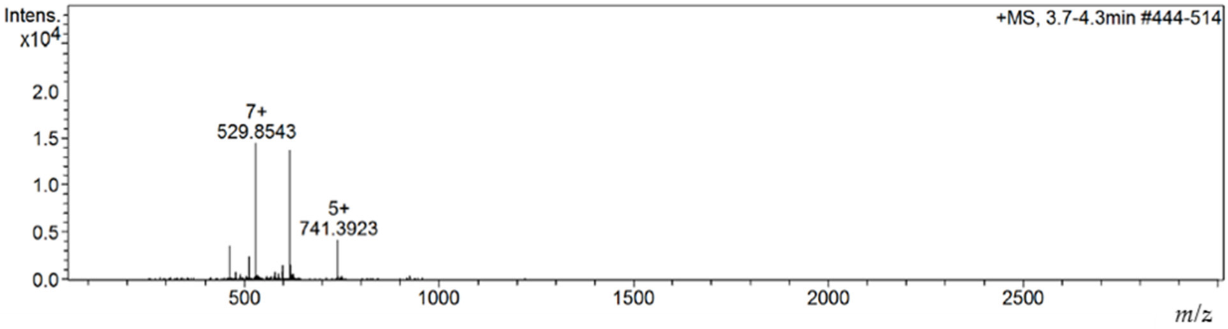

Figure S16. HRMS chromatogram of oxidized DV1-k-(DV3).

1.8. [natGa]PentixaFor

A solution containing 2 mM of PentixaFor acetate (ABX advanced biochemical compounds GmbH, Radeberg, Germany, 250 µg in 100 µL, 1 eq.) and $Ga(NO_3)_3$ (10 eq.) in metal-metal free water was prepared. The reaction mixture was allowed to react for 30 min at 95 °C. Complete conversion to [natGa]PentixaFor was confirmed by LC-HRMS analysis. The solution was used as such for further in vitro half-maximal inhibitory concentration (IC_{50}) determination. $C_{60}H_{78}N_{14}O_{14}Ga$ theoretical neutral average mass (ESI-LCMS) 1289.07 Da; found 1288.72 ± 0.01 Da.

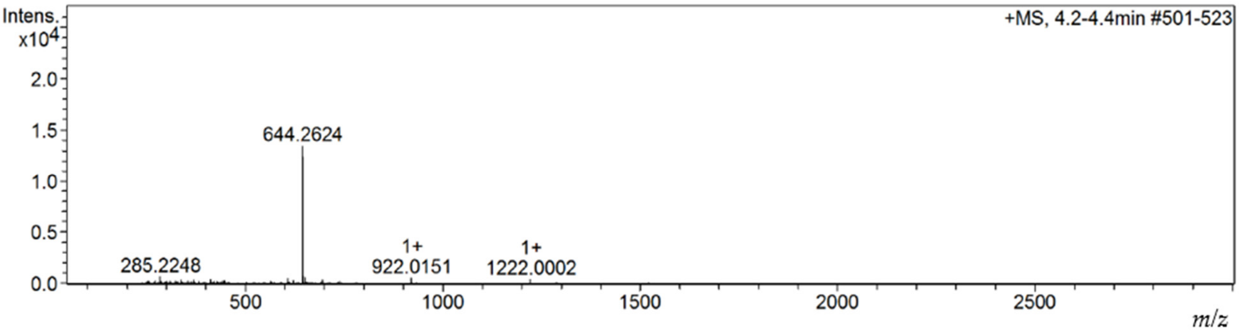

Figure S17. HRMS chromatogram of [natGa]PentixaFor.

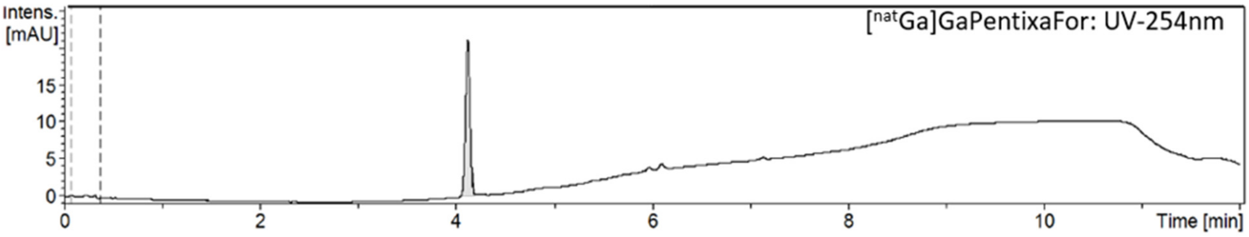

Figure S18. HPLC chromatogram of [natGa]PentixaFor (UV-254nm).

2. Radiosynthesis

2.1. [18F]AlF-NOTA-DV1-k-(DV3)

Fluorine-18 was produced on site using a cyclotron (IBA Cyclone 18/9, IBA, Louvain-la-Neuve, Belgium) by irradiation of $H_2[^{18}O]O$ with 18-MeV protons.

To the precursor solution containing 120 nmol NOTA-DV1-k-(DV3) in 0.1 M sodium acetate pH 4.1 (0.3 mL) and EtOH (0.3 mL), 30 µL of a freshly prepared sodium ascorbate solution (20 mg/mL sodium ascorbate (Ph. Eur, Fagron, Nazareth, Belgium) in HPCE grade water (Sigma Aldrich, Saint Louis, MO, USA)) was added just before the radiolabeling. All buffers used for radiolabeling were treated with chelex (chelex, 100 sodium form (Sigma Aldrich, Saint Louis, MO, USA), 2 g/L, 30 min stirring at room temperature and filtration with a 0.45 µm polyamide filter (Sartorius Stedim Biotech, Göttingen, Germany)).

[18F]AlF-NOTA-DV1-k-(DV3) was synthesized in an AllInOne® synthesis module (Trasis, Ans, Belgium). During the placement of vials and reagents on the cassette, the 4 mL cyclic olefin copolymer reactor was prefilled with 25 µL of 2 mM aluminum chloride ($AlCl_3$, anhydrous, powder, 99.999% trace metals basis, Sigma-Aldrich, Saint Louis, MO, USA) in sodium acetate buffer (0.1 M, pH 4.1). [18F]fluoride (8 GBq) was transferred to the module and trapped on a Sep-Pak light Accel plus anion exchange cartridge (Cl^- form: Waters Corporation, Milford, MA, USA). The cartridge was washed with 6 mL of water

(HPCE grade, Sigma Aldrich, Saint Louis, MO, USA). [18F]fluoride was eluted from the QMA cartridge into a reservoir (5 mL Inject syringe; BBraun, Melsungen, Germany) with 500 µL of the eluent solution consisting out of 250 µL NaCl 0.9% (99.999% trace metals basis NaCl (Sigma Aldrich, Saint Louis, MO, USA) in HPCE grade water (Sigma Aldrich, Saint Louis, MO, USA)) and 250 µL EtOH. 250 µL of the [18F]fluoride containing eluate was transferred to the reactor containing the aluminum chloride solution. The solution was stirred for 2 min at room temperature under gentle nitrogen flow (N_2) to form [18F]AlF. The precursor solution (600 µL of 0.2 mg/mL NOTA-DV1-k-(DV3) and 0.95 mg/mL sodium ascorbate in sodium acetate 0.1 M pH 4.1/EtOH (50/50 *v*/*v*)) was added to the reactor, which was sealed and heated for 10 min at 100 °C. Around 100 µL of precursor solution is left in the precursor vial after transfer to the reactor. Next, the reactor was cooled to 40 °C, the reaction mixture was transferred to a dilution vial filled with 15 mL formulation solution (sodium ascorbate 0.59% in NaCl 0.9% in water for injection) and mixed under gentle N_2 flow. The diluted solution was transferred over a Sep-Pak light C_{18} cartridge (Waters Corporation, Milford, MA, USA), which was preconditioned with 5 mL EtOH and 10 mL water. Afterwards, the cartridge was washed with 20 mL formulation solution and flushed with N_2 to remove free [18F]fluoride and unreacted ([18F]AlF)$^{2+}$. [18F]AlF-NOTA-DV1-k-(DV3) was eluted from the SPE cartridge to the dispensing cell with 1.6 mL EtOH and the SPE cartridge was flushed with 17.4 mL of the formulation solution. The eluate was passed through a 0.22 µm sterile filter (Millex-GV, 0.22 µm, PVDF, 13 mm, Merck KGaA, Darmstadt, Germany) into a sterile 25 mL dose vial. The final drug product solution ([18F]AlF-NOTA-DV1-k-(DV3) in EtOH/sodium ascorbate 0.59% in NaCl 0.9% in water for injection) was measured in an ionization chamber-based activity meter (COMECER VIK-203, Comecer S.p.A., Castel Bolognese, Italy) and samples were taken for quality control [2]. Specific activity was determined based on the concentration of peptide at the start of the reaction (11.5 ± 2.5 MBq/nmol, *n* = 3). Radiochemical purity was determined by radioHPLC. Recovery rates of [18F]F$^-$: 113 ± 2%; ([18F]AlF)$^{2+}$: 100 ± 5%; [18F]AlF-NOTA-DV1-k-(DV3): 93 ± 6%.

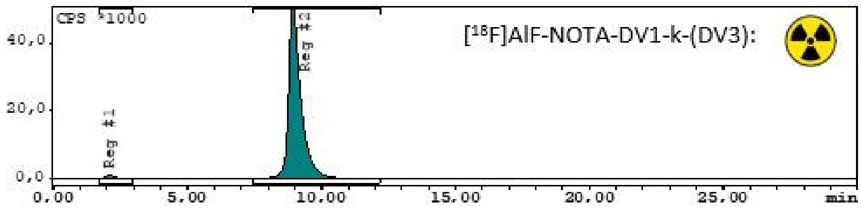

Figure S19. RadioHPLC chromatogram of [18F]AlF-NOTA-DV1-k-(DV3).

2.2. *[68Ga]Ga-DOTA-DV1-k-(DV3) and [68Ga]PentixaFor*

[68Ga]Ga-DOTA-DV1-k-(DV3) and [68Ga]PentixaFor were produced using a SCINTOMICS GRP module in combination with a disposable GMP grade cassette system (ABX advanced biochemical compounds GmbH, Radeberg, Germany). All reagents were carefully selected to minimize presence of metal contaminants, e.g. iron. Gallium-68 was eluted in the form of [68Ga][$GaCl_4$]$^-$ from a commercially available IGG101 Pharmaceutical Grade Generator from Eckert & Ziegler (Berlin, Germany) using 0.1 M HCl. To concentrate gallium-68 and remove any germanium-68 in the generator eluate, the mixture was applied on a Chromafix PS-H$^+$ column. Gallium-68 was eluted with 1.5 mL of 5 M sodium chloride into the reactor vial containing 40 nmol DOTA-DV1-k-(DV3) or 17 nmol PentixaFor acetate (ABX advanced biochemical compounds GmbH, Radeberg, Germany) in 3 mL of 1.5 M HEPES. The radiolabeling mixture was heated for 10 min at 95 °C (DOTA-DV1-k-(DV3) or 140 °C (PentixaFor). After cooling, the reaction mixture was passed over a Sep-Pak C_{18} Light cartridge (Waters Corporation, Milford, MA, USA). The Sep-Pak C_{18}

Light cartridge was pre-conditioned with EtOH (5 mL) and water for injection (10 mL). The radiolabeled peptide was eluted from the cartridge with 1 mL EtOH (DOTA-DV1-k-DV3) or 2 mL EtOH/water for injection (50:50 (*v*/*v*), PentixaFor) and diluted with sodium ascorbate 0.59% in NaCl 0.9% in water for injection or PBS (EtOH content below 8%). Finally, sterile filtration was performed using a vented 0.22 µm filter (Cathivex Merck Millipore). Specific activity was determined based on the concentration of peptide at the start of the reaction ([^{68}Ga]Ga-DOTA-DV1-k-(DV3) 8.9 ± 1.2 MBq/nmol (*n* = 5), [^{68}Ga]PentixaFor 29.2 ± 3.7 MBq/nmol (*n* = 3)). Radiochemical purity was determined by radioHPLC.

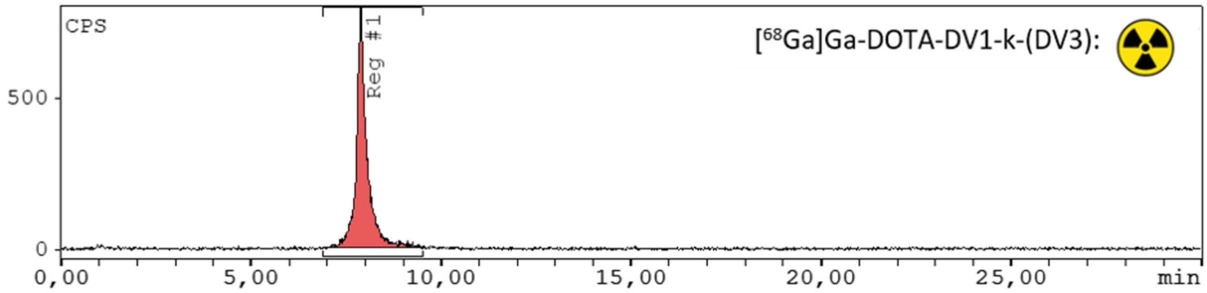

Figure S20. RadioHPLC chromatogram of [^{68}Ga]Ga-DOTA-DV1-k-(DV3).

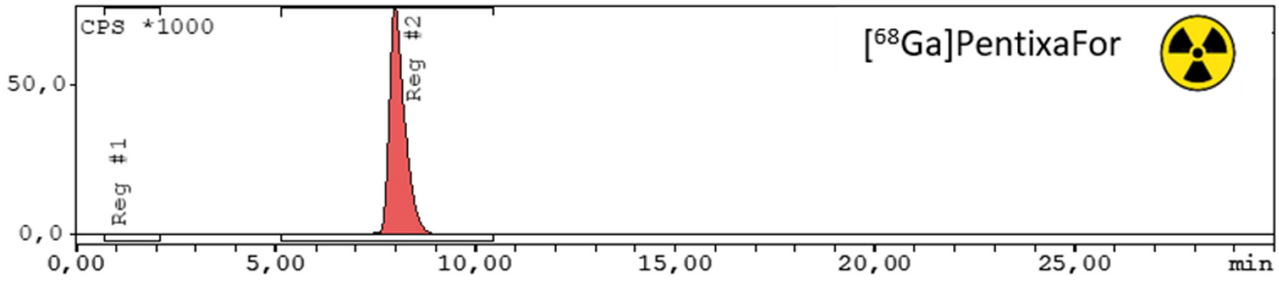

Figure S21. RadioHPLC chromatogram of [^{68}Ga]PentixaFor.

2.3. [^{177}Lu]Lu-DOTA-DV1-k-(DV3)

A solution containing DOTA-DV1-k-(DV3) (10 µM in 0.1 M sodium acetate, pH 4.1) and [^{177}Lu]$LuCl_3$ (50 MBq in 0.01 N HCl, ITM Isotopen Technologien Munchen Gmbh, Garching-Munich, Germany) in a total volume of 500 µL was prepared. The reaction mixture was allowed to react for 15 min at 95 °C. After the reaction, a sample was taken for quality control, iTLC and radioHPLC analysis, before formulating the final drug product in sodium ascorbate 0.59%, NaCl 0.9% in water for injection and sterile filtration through a 0.22 µm sterile filter (Millex-GV, 0.22 µm, PVDF, 13 mm, Merck KGaA, Darmstadt, Germany). Specific activity was determined based on the concentration of peptide at the start of the reaction ([^{177}Lu]Lu-DOTA-DV1-k-(DV3) 12.5 MBq/nmol (*n* = 2)). Radiochemical purity was determined by radioHPLC.

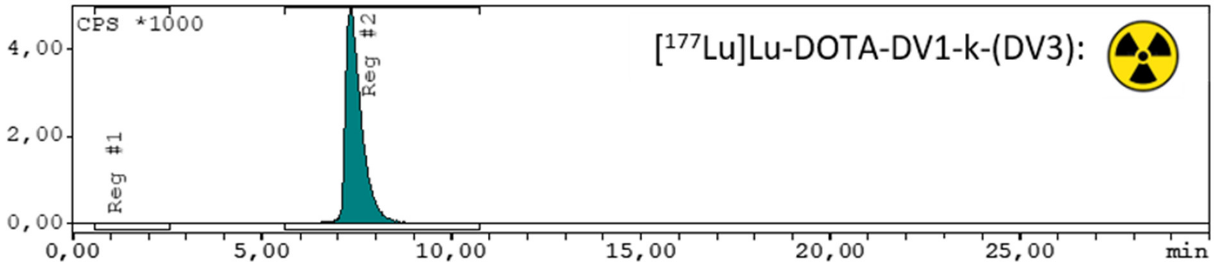

Figure S22. RadioHPLC chromatogram of [^{177}Lu]Lu-DOTA-DV1-k-(DV3).

3. Biological Experiments

3.1. In vitro Stability

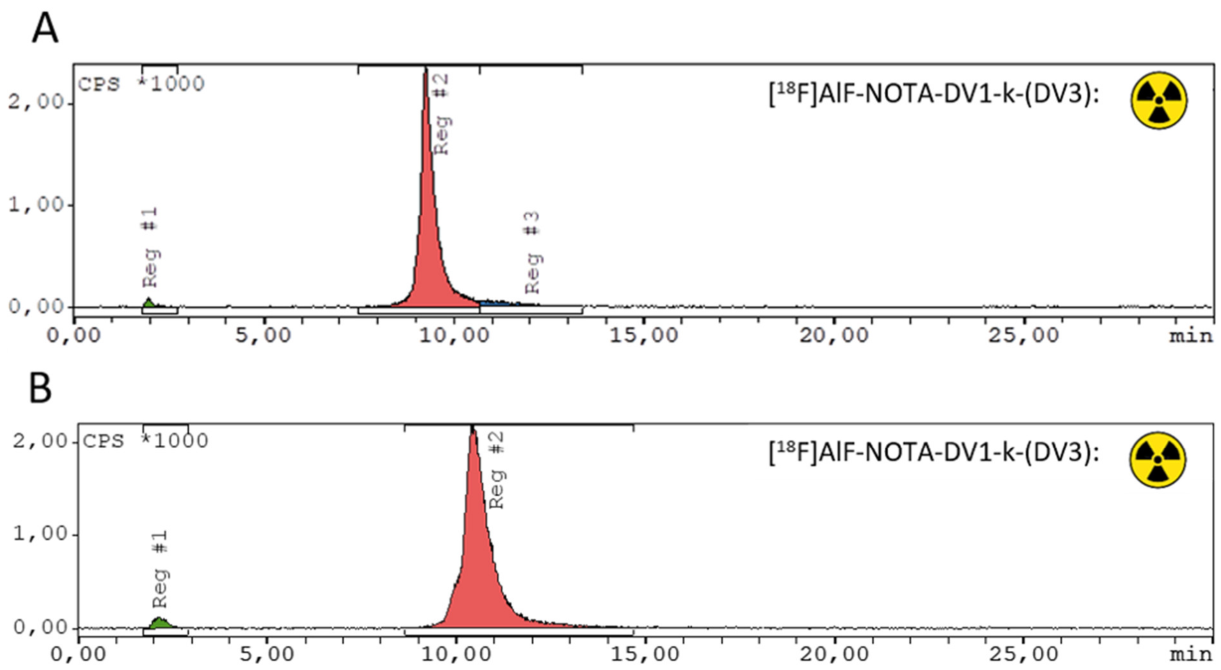

Figure S23. In vitro stability study of [18F]AlF-NOTA-DV1-k-(DV3) in formulation buffer (**A**, room temperature, 5 h) and human serum (**B**, 37 °C, 2 h).

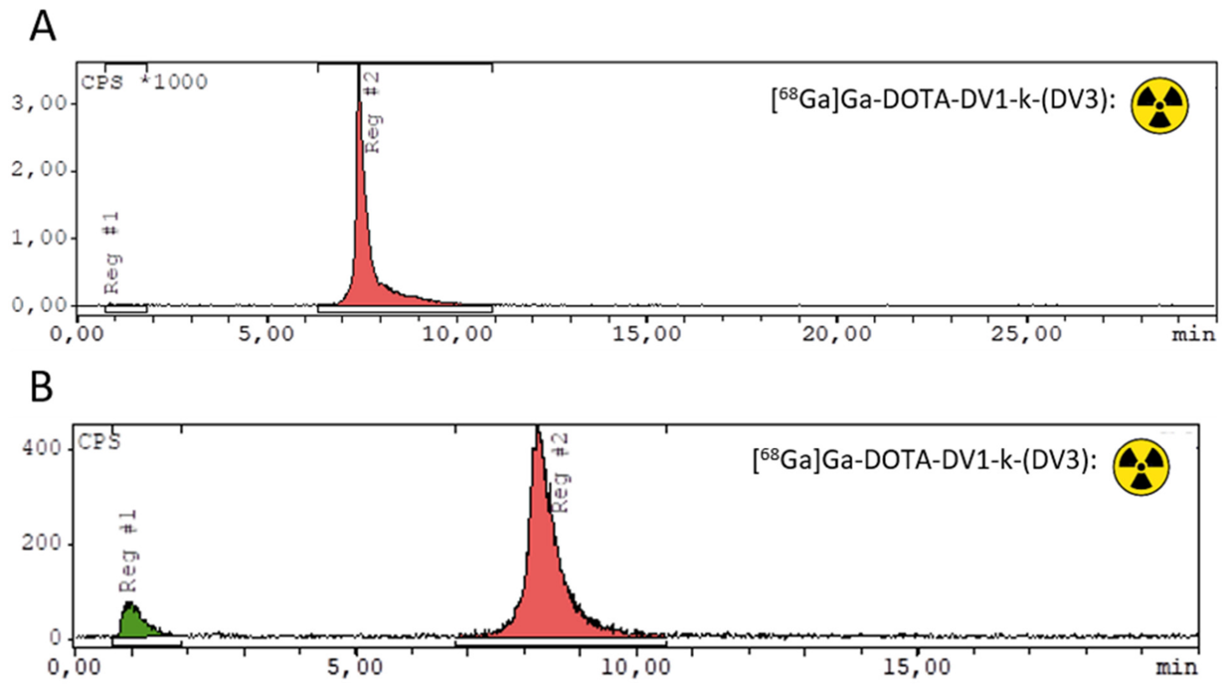

Figure S24. In vitro stability study (2 h) of [68Ga]Ga-DOTA-DV1-k-(DV3) in formulation buffer (**A**, room temperature) and human serum (**B**, 37 °C).

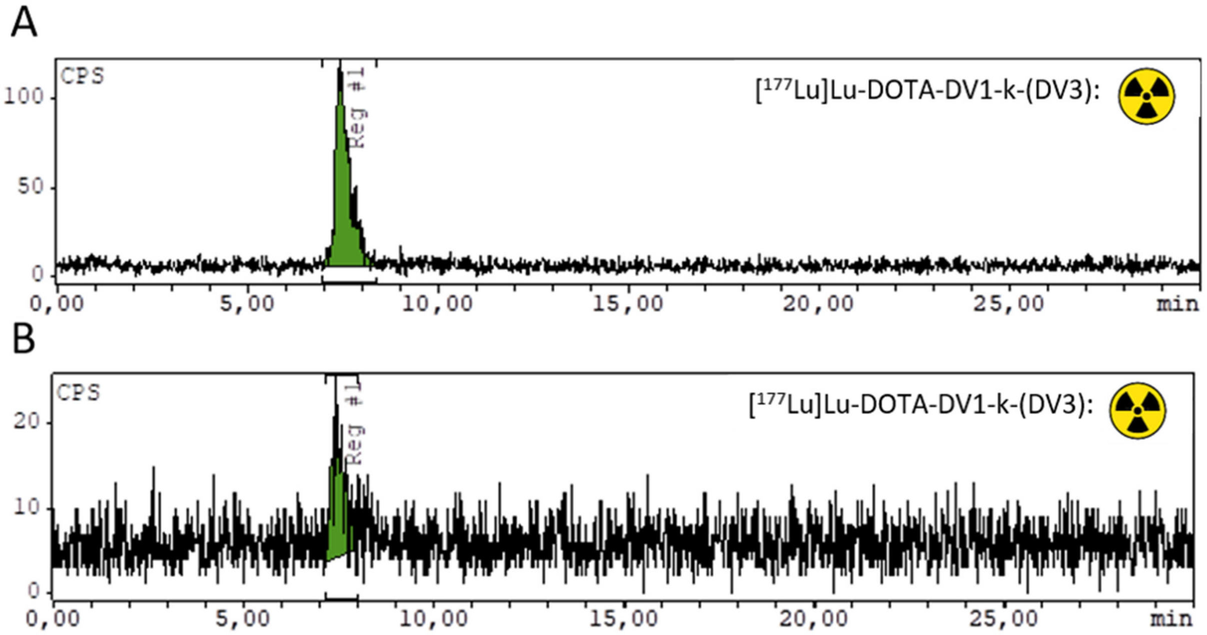

Figure S25. In vitro stability study (9 days) of [^{177}Lu]Lu-DOTA-DV1-k-(DV3) in formulation buffer (**A**, 4 °C) and human serum (**B**, 37 °C).

3.2. Ex vivo Biodistribution in Wild-type Mice

Table S2. Ex vivo biodistribution in wild-type mice 75 min p.i. (expressed in SUV). [^{68}Ga]PentixaFor (*n* = 3), [^{68}Ga]PentixaFor + 5 mg/kg AMD3100 (*n* = 1), [^{18}F]AlF-NOTA-DV1-k-(DV3) (*n* = 5), [^{18}F]AlF-NOTA-DV1-k-(DV3) + 5 mg/kg AMD3100 (*n* = 2), [^{68}Ga]Ga-DOTA-DV1-k-(DV3) (*n* = 4), [^{68}Ga]Ga-DOTA-DV1-k-(DV3) + 5 mg/kg AMD3100 (*n* = 4), [^{177}Lu]Lu-DOTA-DV1-k-(DV3) (*n* = 3) and [^{177}Lu]Lu-DOTA-DV1-k-(DV3) + 5 mg/kg AMD3100 (*n* = 3). Statistical analysis independent-samples t-test. All data are expressed as mean ± SD. * $p < 0.05$, ** $p \leq 0.01$, *** $p \leq 0.001$.

	**[^{68}Ga]PentixaFor**		**[^{18}F]AlF-NOTA-DV1-k-(DV3)**		**[^{68}Ga]Ga-DOTA-DV1-k-(DV3)**		**[^{177}Lu]Lu-DOTA-DV1-k-(DV3)**	
**AMD3100**		**5 mg/kg**		**5 mg/kg**		**5 mg/kg**		**5 mg/kg**
	n = 3	*n* = 1	*n* = 5	*n* = 2	*n* = 4	*n* = 4	*n* = 3	*n* = 3
kidneys	1.9 ± 1.1	1.20	23.2 ± 3.9	35.4	44.0 ± 10.2	46.7 ± 17.8	35.8 ± 3.1	71.4 ± 5.8***
liver	0.6 ± 0.1	0.50	7.4 ± 0.5	1.0	9.8 ± 0.6	1.3 ± 0.4***	7.5 ± 0.7	0.5 ± 0.04***
spleen	0.3 ± 0.02	0.30	3.2 ± 0.8	0.9	2.6 ± 1.4	0.9 ± 0.4*	1.3 ± 0.4	0.2 ± 0.01*
pancreas	0.3 ± 0.1	0.30	0.1 ± 0.02	0.1	0.1 ± 0.02	0.3 ± 0.1	0.1 ± 0.03	0.1 ± 0.01
lungs	0.7 ± 0.1	0.70	0.8 ± 0.2	0.8	0.3 ± 0.1	0.9 ± 0.5	0.1 ± 0.01	0.2 ± 0.02
heart	0.3 ± 0.02	0.30	0.2 ± 0.1	0.2	0.1 ± 0.02	0.4 ± 0.3	0.04 ± 0.01	0.1 ± 0.01
brain	0.03 ± 0.01	0.02	0.01 ± 0.003	0.02	0.04 ± 0.06	0.03 ± 0.02	0.003 ± 0.002	0.004 ± 0.002
blood	0.6 ± 0.1	0.50	0.2 ± 0.1	0.5	0.1 ± 0.02	0.1 ± 0.1	0.03 ± 0.003	0.1 ± 0.01**
bone	0.2 ± 0.01	0.20	0.7 ± 0.4	0.5	0.4 ± 0.1	0.3 ± 0.2	0.5 ± 0.04	0.1 ± 0.04***
muscle	0.2 ± 0.03	0.20	0.1 ± 0.04	0.1	0.05 ± 0.02	0.2 ± 0.1*	0.04 ± 0.01	0.02 ± 0.02

3.3. Ex vivo Autoradiography of Kidney, Liver and Spleen of Wild-type Mice

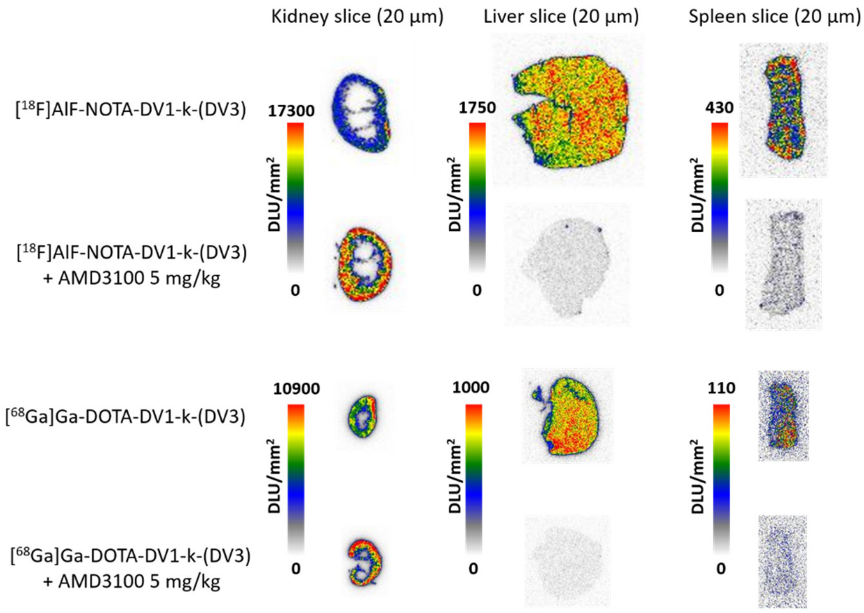

Figure S26. Ex vivo autoradiography of kidney, liver and spleen of wild-type mice 75 min p.i. of [^{18}F]AlF-NOTA-DV1-k-(DV3), [^{18}F]AlF-NOTA-DV1-k-(DV3) + 5 mg/kg AMD3100, [^{68}Ga]Ga-DOTA-DV1-k-(DV3) and [^{68}Ga]Ga-DOTA-DV1-k-(DV3) + 5 mg/kg AMD3100.

3.4. *In vivo stability*

Plasma	15 min p.i.
	n = 2
Polar metabolites	14 ± 0.1%
Intact tracer	86 ± 0.1%

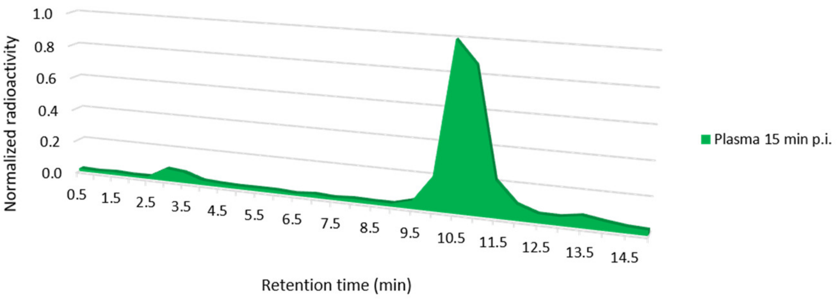

Figure S27. In vivo radiometabolite study of [^{18}F]AlF-NOTA-DV1-k-(DV3) in plasma of wild-type mice 15 min p.i..

Urine	75 min p.i.
	n = 3
Polar metabolites	63 ± 2%
Intact tracer	37 ± 2%

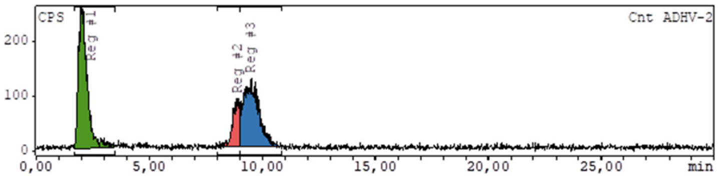

Figure S28. In vivo radiometabolite study of [^{18}F]AlF-NOTA-DV1-k-(DV3) in urine of wild-type mice 75 min p.i..

3.5. Ex vivo biodistribution in U87.hCXCR4 tumor-bearing mice

Table S3. Ex vivo biodistribution in U87.hCXCR4 tumor-bearing mice 75 min p.i. (expressed in SUV). Statistical analysis independent-samples t-test. All data are expressed as mean ± SD. * $p < 0.05$, ** $p \leq 0.01$, *** $p \leq 0.001$.

	[^{18}F]AlF-NOTA-DV1-k-(DV3)				
**AMD3100**	**0 nmol** *n* = 4				**157 nmol** *n* = 4
**DV1-k-(DV3)**		**7 nmol** *n* = 3	**14 nmol** *n* = 4	**24 nmol** *n* = 4	
kidneys	26.5 ± 6.0	39.2 ± 7.6*	33.6 ± 12.4	36.1 ± 5.9*	47.5 ± 3.1***
liver	8.2 ± 1.0	1.2 ± 0.1***	1.2 ± 0.4***	0.7 ± 0.1***	0.9 ± 0.3***
spleen	2.5 ± 1.0	0.5 ± 0.1**	0.6 ± 0.2*	0.4 ± 0.1**	0.5 ± 0.1**
pancreas	0.06 ± 0.02	0.1 ± 0.04	0.2 ± 0.1	0.1 ± 0.03	0.1 ± 0.1
lungs	0.6 ± 0.2	0.7 ± 0.2	0.9 ± 0.3	0.6 ± 0.2	0.5 ± 0.1
heart	0.1 ± 0.04	0.2 ± 0.1	0.3 ± 0.1	0.2 ± 0.1	0.1 ± 0.1
brain	0.01 ± 0.001	0.02 ± 0.01	0.03 ± 0.01	0.02 ± 0.01	0.01 ± 0.004
bone	0.4 ± 0.1	0.2 ± 0.04**	0.3 ± 0.1*	0.2 ± 0.04**	0.1 ± 0.04**
tumor	0.6 ± 0.2	0.7 ± 0.1	0.9 ± 0.3	0.6 ± 0.2	0.4 ± 0.2
muscle	0.05 ± 0.02	0.2 ± 0.1	0.2 ± 0.1*	0.2 ± 0.1*	0.1 ± 0.1
blood	0.1 ± 0.1	0.4 ± 0.2	0.5 ± 0.3*	0.3 ± 0.1*	0.3 ± 0.2
t/liver	0.1 ± 0.02	0.6 ± 0.1**	0.8 ± 0.2**	0.9 ± 0.2***	0.5 ± 0.2*
t/spleen	0.3 ± 0.1	1.4 ± 0.1***	1.4 ± 0.1***	1.6 ± 0.3***	0.7 ± 0.2**
t/bone	1.4 ± 0.5	2.9 ± 0.3**	3.2 ± 0.4***	3.3 ± 0.5***	2.6 ± 0.7*
t/muscle	12.4 ± 2.9	5.4 ± 2.2**	4.8 ± 2.1**	3.0 ± 0.4**	4.2 ± 1.1**
t/blood	4.6 ± 0.9	2.2 ± 0.9**	2.0 ± 0.9**	1.8 ± 0.3**	1.7 ± 0.5***

References

1. Suzuki, K.; Ui, T.; Nagano, A.; Hino, A.; Arano, Y. C-terminal–modified LY2510924: a versatile scaffold for targeting C-X-C chemokine receptor type 4. *Sci. Rep.* **2019**, *9*, 1–10, doi:10.1038/s41598-019-51754-0.
2. Tshibangu, T.; Cawthorne, C.; Serdons, K.; Pauwels, E.; Gsell, W.; Bormans, G.; Deroose, C.M.; Cleeren, F. Automated GMP compliant production of [18F]AlF-NOTA-octreotide. *EJNMMI Radiopharm Chem.* **2020**; 5, 1–23.
